# Temperature Coefficients of Perovskite Photovoltaics for Energy Yield Calculations

**DOI:** 10.1021/acsenergylett.1c00748

**Published:** 2021-05-07

**Authors:** Taylor Moot, Jay B. Patel, Gabriel McAndrews, Eli J. Wolf, Daniel Morales, Isaac E. Gould, Bryan A. Rosales, Caleb C. Boyd, Lance M. Wheeler, Philip A. Parilla, Steven W. Johnston, Laura T. Schelhas, Michael D. McGehee, Joseph M. Luther

**Affiliations:** † National Renewable Energy Laboratory, Golden Colorado 80401, United States; ‡ Department of Chemical and Biological Engineering, University of Colorado, Boulder, Colorado 80309, United States; § Materials Science and Engineering, University of Colorado, Boulder, Colorado 80309, United States; ∥ Department of Applied Physics, Stanford University, Stanford, California 94305, United States; ⊥ Department of Materials Science and Engineering, Stanford University, Stanford, California 94305, United States

## Abstract

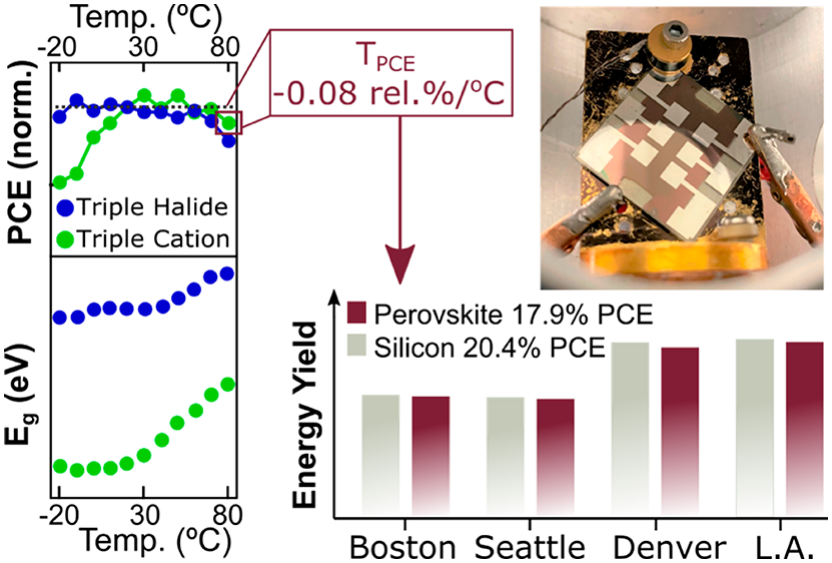

Temperature coefficients for maximum power (*T*_PCE_), open circuit voltage (*V*_OC_), and short circuit current (*J*_SC_) are standard specifications included in data sheets for any commercially available photovoltaic module. To date, there has been little work on determining the *T*_PCE_ for perovskite photovoltaics (PV). We fabricate perovskite solar cells with a *T*_PCE_ of −0.08 rel %/°C and then disentangle the temperature-dependent effects of the perovskite absorber, contact layers, and interfaces by comparing different device architectures and using drift-diffusion modeling. A main factor contributing to the small *T*_PCE_ of perovskites is their low intrinsic carrier concentrations with respect to Si and GaAs, which can be explained by its wider band gap. We demonstrate that the unique increase in *E*_g_ with increasing temperatures seen for perovskites results in a reduction in *J*_SC_ but positively influences *V*_OC_. The current limiting factors for the *T*_PCE_ in perovskite PV are identified to originate from interfacial effects.

The exceptional laboratory research progress made on perovskite photovoltaics (PV) has led to remarkably high power conversion efficiencies (PCE), reaching 25.5% for single junctions and 29.1% for perovskite–Si tandems,^1^ which rival the champion efficiencies of GaAs or Si.^2^ Major advances in device operational stability have been made through the careful control of interfaces, contact layers, and metal–halide perovskite (referred to as perovskites in this study) compositions with many reports showing greater than 1000 h of stability at elevated temperatures, reaching the critical testing milestones outlined in ISOS or IEC protocols.^3−6^ Finally, a 17.9% efficient module with area > 800 cm^2^ has been reported, which is close to the PCE of currently commercialized CIGS and CdTe technologies although the modules do not yet demonstrate the same level of stability.^7^

Despite this remarkable progress and trajectory toward various commercial marketplaces,^8^ less attention has been paid to the device performance under variable operational conditions, namely, at elevated temperatures and nonstandard 1 sun testing conditions, as would be experienced from off-angle irradiation or cloud coverage.^9−13^ Understanding how these conditions affect the performance during operation is critical for predicting the energy yield, which is a key metric to any technoeconomic analysis.^14,15^ The increase in perovskite solar cell stability has resulted in reports of the real time energy yield of perovskite solar cells, with the solar cells showing remarkable potential for terrestrial applications.^11^ A key proxy for energy yield that is reported is the PCE temperature coefficient (*T*_PCE_), which is equal to the change in PCE divided by the change in temperature when the photovoltaic is operated at variable temperatures as shown in eq 1.^11,16,17^
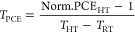
1where Norm.PCE_HT_ is the PCE of a solar cell at a higher temperature normalized with respect to the room-temperature PCE, *T*_HT_ is the temperature of the cell at higher temperature, and *T*_RT_ is the temperature of the cell at room temperature.

For terrestrial utility-scale PV applications, the typical operating temperature range is approximately −20 to 85 °C;^18^ thus, we focus on the high-temperature *T*_PCE_. Even after only a handful of reports, it is clear that perovskite PV have a much smaller (better) *T*_PCE_ at elevated temperatures,^9,11,19,20^ reaching a champion of ∼−0.13 rel %/°C.^19^ However, the origin of these remarkably low values is not well understood.

There are a few hypotheses to explain the low perovskite *T*_PCE_. First, a key and important factor that will yield a lower *T*_PCE_ is because popular perovskite compositions have a wider band gap than current mainstream technologies; thus, the increase in dark carrier density with temperature and the resulting increase in the dark saturation current are less. Second, the band gap (*E*_g_) of perovskites increases with temperature,^21−23^ opposite from almost all other traditional semiconductors. Supporting Information Figure S1 shows a graphical representation of *E*_g_ vs temperature for select references to illustrate this point.^24^ The increase in *E*_g_ could mitigate losses in *V*_OC_, directly impacting PCE. Dupre et al. have predicted that the characteristic band gap blue shift of perovskite semiconductors at elevated temperatures can have implications in the magnitude of the *T*_PCE_ on the basis of how far and which direction the room-temperature band gap is from the optimal single junction band gap (1.4 eV).^25^ On the other hand, Aydin et al. have recently shown that to maximize the PCE of multijunction Si–perovskite solar cells at elevated operating temperatures it is critical to tailor the perovskite band gap to account for their increasing *E*_g_ shift, mitigating the current mismatch that may arise at elevated temperatures otherwise.^26^ Thus, the role of the perovskite *E*_g_ shift has implications on the *JV* metrics for both single junction and multijunction devices. Third, perovskites have a uniquely high defect tolerance, potentially mitigating any deleterious effects of an increase in available defect states^27^ by minimizing impacts of recombination rates,^22,28^ mobility,^22^ dielectric constant,^29^ or interfacial phenomena, including changes in surface recombination.^30^ Nevertheless, there are also possible adverse effects on the *T*_PCE_, including changes in contact resistance,^31^ energetic alignment,^16,32^ or issues with perovskite device stability at elevated operating temperatures.^9^ While these hypotheses are all plausible, it is difficult to discern which ones are correct because nearly all work on *T*_PCE_ utilized MAPbI_3_ (MA = methylammonium) and/or the device architecture TiO_2_/perovskite/spiro-OMeTAD/Au.^16,17,19,20,33−36^ The heavy focus on a singular device architecture makes it difficult to understand how the solar cell architecture and device instability all affect the *T*_PCE_.^16,17,33−35^

Here, we disentangle how the perovskite, contact layers and interfaces each affect the *T*_PCE_ through comparison between four different device architectures. We minimize the influence of device instability by choosing perovskite compositions and device architectures with state-of-the-art stability and measure a *T*_PCE_ of up to −0.08 rel %/°C, and then we delve into elucidating the basis behind the low *T*_PCE_. From this, we identify that the perovskite composition exclusively controls changes in short circuit current density (*J*_SC_) vs temperature and that the specific perovskite/contact layer interface controls open circuit voltage (*V*_OC_) and fill factor (FF). These conclusions are explained with drift-diffusion modeling. We then calculate the energy yield for real world operating conditions, by inputting both the measured temperature and illumination PCE dependence of both perovskite and Si devices, assuming current record module efficiencies.

We focus on p–i–n architecture perovskite solar cells due to their high efficiency, stability, and regular use in perovskite-based tandems. Two perovskite compositions were chosen (FA= formamidinium): (1) FA_0.79_MA_0.16_Cs_0.05_Pb(I_0.83_,Br_0.17_)_3_ (colloquially referred to as “triple cation”) because of its ubiquity throughout the perovskite field, robust stability, and use in the champion 23.3% perovskite/CIGS tandem^37−39^ and (2) FA_0.75_Cs_0.22_MA_0.03_Pb(I_0.82_Br_0.15_Cl_0.03_)_3_ (colloquially referred to as “triple halide”) because of its use in a 27% perovskite/silicon tandem with exceptional operational stability.^40,41^ Two hole transport layers (HTLs), NiO_*x*_ and PTAA, were chosen due to their stability, high efficiency, and use in perovskite, CIGS, and/or Si–perovskite tandems.^40,42,43^ The devices were completed with thermally evaporated LiF/C_60_/BCP/Ag and reached an average ∼17% PCE when measured under 1 sun at room temperature in N_2_ (Figure S2). Device performance at operational temperatures (Figure 1) was measured from −20 to 80 °C, representative of the range of potential operating temperatures for terrestrial applications, and the average PCE (from reverse curves) from multiple devices was calculated and normalized to room-temperature (20 °C) PCE for comparison. By affixing a thermocouple directly onto the perovskite solar cell, (active layer side), we were able to deduce the effects of heating due to the illumination and found that the solar cells did not heat up from a standard *JV* scan. Further information on our setup and calibration steps can be found in the Supporting Information.

**Figure 1:**
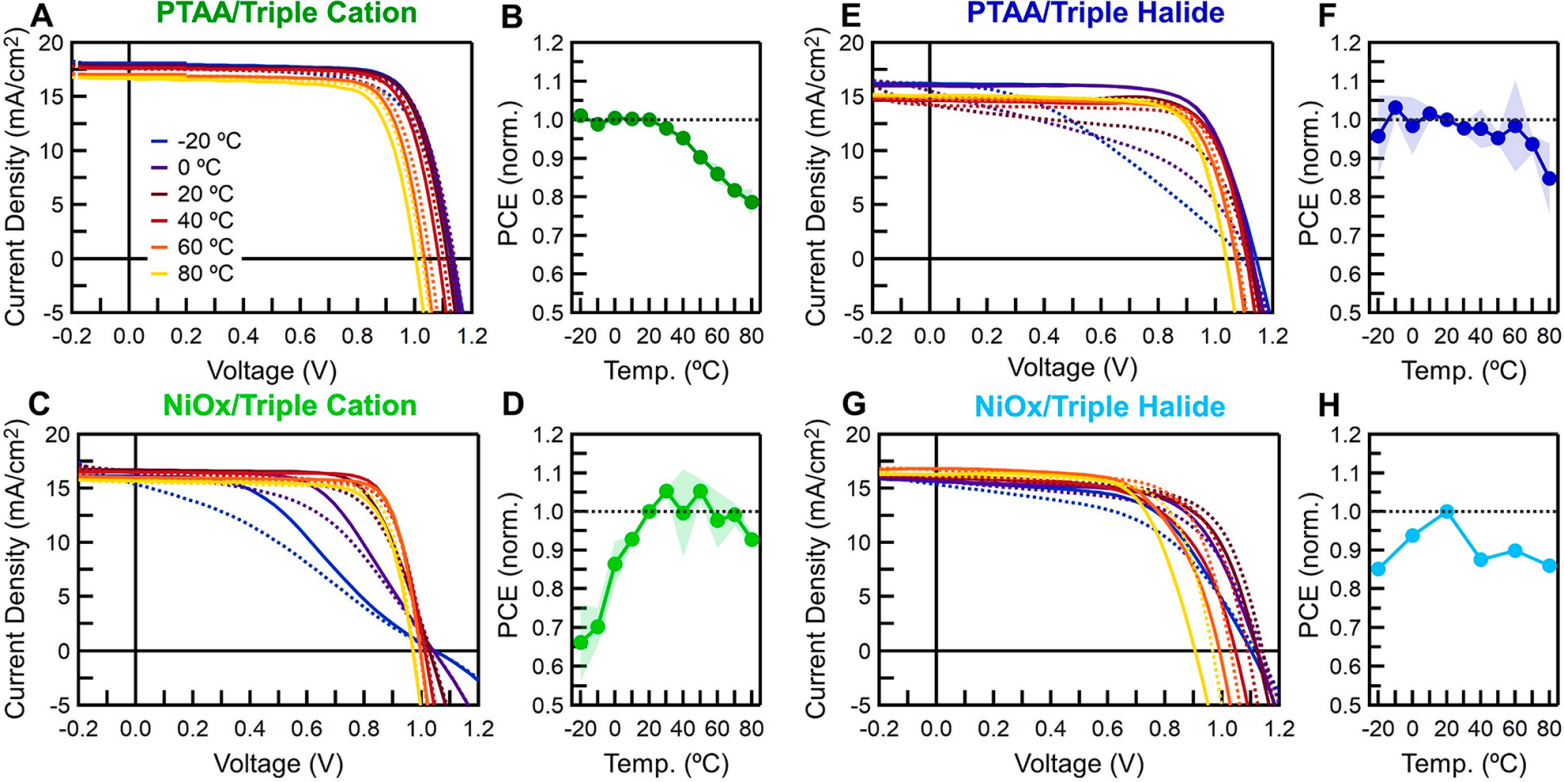
*JV* curves (forward, dashed; reverse, solid) and normalized average PCE with standard deviation (shaded) vs temperature for (A, B) PTAA/triple cation, (C, D) NiO_*x*_/triple cation, (E, F) PTAA/triple halide, and (G, H) NiO_*x*_/triple halide, respectively. Triple cation refers to FA_0.79_MA_0.16_Cs_0.05_Pb(I_0.83_,Br_0.17_)_3_, and triple halide refers to FA_0.75_Cs_0.22_MA_0.03_Pb(I_0.82_Br_0.15_Cl_0.03_)_3_.

First, comparison of PTAA/triple cation and NiO_*x*_/triple cation devices demonstrates a clear difference in the normalized PCE vs temperature trends. For PTAA/triple cation, there is a drop in PCE at elevated temperatures to an average of 79% of starting efficiencies, translating to an average *T*_PCE_ of −0.36 rel %/°C at 80 °C. Conversely, NiO_*x*_/triple cation shows a precipitous PCE drop at colder temperatures down to 66% (which we concur should also be better understood) but a minimal drop in PCE at elevated temperatures, maintaining 93% of the starting efficiency resulting in an average *T*_PCE_ of −0.12 rel %/°C and a champion *T*_PCE_ at of −0.08 rel %/°C at 80 °C. Unlike the triple cation devices, both PTAA/triple halide and NiO_*x*_/triple halide show a drop in PCE at elevated temperatures to an average 85% (−0.25 rel %/°C) and 79% (−0.35 rel %/°C), respectively. Clearly, each HTL/perovskite device has a unique change in PCE at operating temperatures, suggesting that neither the perovskite composition nor the HTL exclusively are responsible for these changes.

The changes in PCE at lower and elevated temperatures are generally reversible (Figure S3) ruling out degradation. Additionally, each device shows increased hysteresis (Figure S4) at low temperatures (<20 °C), previously attributed to decreased ion mobility.^17,32^ The origin of the change in PCE is also different (Figures S5 and S6) between each device architecture. For example, changes in *J*_SC_, *V*_OC_, and FF all contribute to the resulting change in PCE for PTAA/triple cation (Figure S5A–C). Conversely, both triple halide device stacks (Figure S5G–L) are mainly influenced by *V*_OC_ and FF changes. Finally, NiO_*x*_/triple cation (Figure S5D–F) is mostly influenced by a change in FF only. Similar variations in the origin of the perovskite PCE change have been previously reported,^19,35^ in stark contrast to Si, CIGS, or CdTe, where the change in PCE is heavily influenced by the *V*_OC_.^44−46^ Thus, the specific perovskite device architecture not only uniquely controls the change in PCE vs temperature but also the origin of these changes in PCE.

The measured *T*_PCE_ of −0.11 rel %/°C for PTAA/triple halide/LiF/C_60_/BCP/Ag and as low as −0.08 rel %/°C for NiO_*x*_/triple cation/LiF/C_60_/BCP/Ag at 80 °C (Figure 2) are lower (better) than the previously reported *T*_PCE_ for any PV technology. This includes the previous perovskite *T*_PCE_ of −0.12 rel %/°C using a TiO_2_/FAMACsPb(I,Br)_3_/Spiro/Au perovskite solar at 60 °C.^9^ These two devices outperform commercialized Si, CIGS, and CdTe and even outperform III–V triple junctions (3J), which are known for their excellent *T*_PCE_.^44−46,49^

**Figure 2:**
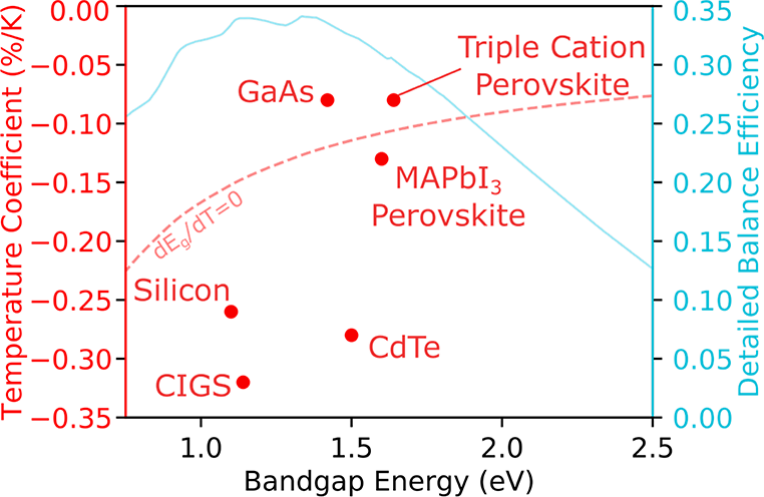
Temperature coefficients of the main photovoltaic technologies overlaid on the detailed balance limit of photovoltaic efficiency at 290 K. The pink dashed line shows the *T*_PCE_ at AM 1.5 and 1 sun and when there is no band gap shift with temperature. A smaller temperature coefficient results in a smaller reduction in power conversion efficiency at higher temperatures. The temperature coefficients were found from the following references: silicon,^47^ CIGS,^46^ GaAs,^48^ CdTe,^45^ MAPbI_3_ perovskite,^19^ and triple cation perovskite (FA_0.79_MA_0.16_Cs_0.05_Pb(I_0.83_,Br_0.17_)_3_) measured in this study. Further details for the different technologies can be found in the Supporting Information.

Next, we performed a detailed study of *J*_SC_ and *V*_OC_ for each device architecture to elucidate the origin of any changes to explain the low-*T*_PCE_ values. The FF in perovskite PV is significantly affected by the hysteresis, and therefore reliably deducing information from it can be nontrivial.^50^ Further analysis on the temperature-dependent changes in the FF can be found in the SI (Note S1). First, we focus on *J*_SC_. Generally, *J*_SC_ is controlled by *E*_g_, absorption coefficient, and charge extraction efficiency. The temperature induced changes in absorbance were measured for triple cation (TC) and triple halide (TH) thin films (Figure 3). Both compositions show an increase in *E*_g_ with increasing temperature;^22^ however, the magnitude of the increase in *E*_g_ is different. Moreover, both compositions show an increase in the lattice constant with increasing temperature. Triple cation, which has a higher fraction of the larger methylammonium (MA) at the A site, has a bigger *E*_g_ shift of 37 meV and a larger initial lattice constant. On the other hand, triple halide has a higher fraction of the smaller Cs at the A site, a smaller *E*_g_ shift of 18 meV, and a smaller initial lattice constant as shown in Figure 3. The perovskite *E*_g_ is determined by the extent of orbital overlap between the antibonding I^–^ and Pb^2+^ orbitals.^51^ As the temperature increases, the perovskite lattice expands (Figure 3), resulting in a decrease in overlap between the I^–^ and Pb^2+^ antibonding orbital, decreasing the valence band energy and consequently increasing the *E*_g_.^23,52,53^ Lattice expansion as a function of temperature is heavily influenced by the exact perovskite composition and the structure it adopts. For example, it is known that at room temperature the incorporation of smaller A-site cations such as MA and Cs not only cause a contraction of the standard 3D perovskite lattice but may also induce octahedral tilting to compensate for the decreased cation size.^54,55^ This suggests that the *magnitude* of the temperature-dependent shift in *E*_g_ for perovskites is a combination of their thermally induced lattice expansion and additional compositionally dependent factors such as the number of electron–phonon interactions at higher temperatures. Ultimately the composition of the perovskite will determine which factors have a greater influence on the temperature-dependent *E*_g_ shift.

**Figure 3:**
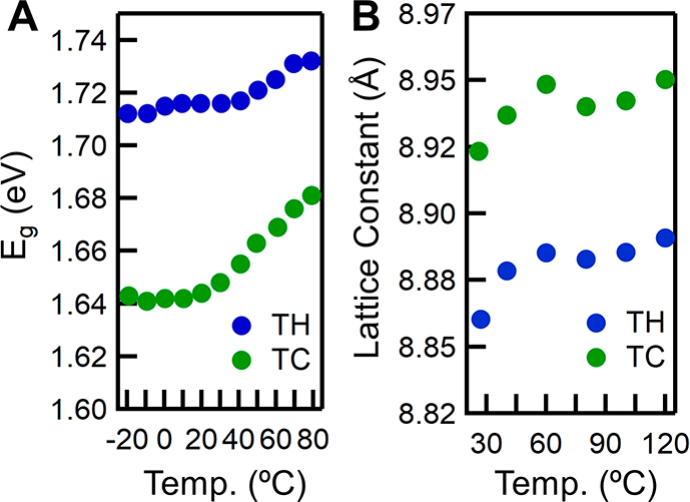
(A) Band gap (*E*_g_) determined using temperature-dependent absorption spectroscopy and (B) lattice constant determined using temperature-dependent X-ray diffraction analysis for both the triple cation (TC), which refers to FA_0.79_MA_0.16_Cs_0.05_Pb(I_0.83_,Br_0.17_)_3_, and triple halide (TH), which refers to FA_0.75_Cs_0.22_MA_0.03_Pb(I_0.82_Br_0.15_Cl_0.03_)_3_, perovskite thin films.

To monitor *E*_g_ changes and the corresponding photovoltaic performance, temperature-dependent external quantum efficiency (EQE) (Figure 4) spectra were taken. The change in EQE onsets matches the temperature-dependent absorbance *E*_g_ shift. EQE can also indicate changes in charge extraction. Suppressed EQE at short or long wavelengths can reveal a temperature-dependent defective interface at the HTL or ETL, respectively. There are no changes in the EQE, except at the absorption onset, indicating no temperature-dependent charge extraction barriers. This may mean that the temperature-dependent changes in the *E*_g_ of all the semiconductors in the device are synchronized and prevent the formation of charge extraction barriers. Moreover, this lack of spectral shape change suggests that the decrease in *J*_SC_ at elevated temperatures is driven by the specific *E*_g_ blue shift and is confirmed in Figure S7. Although the blue shift is larger in triple cation than triple halide, there is a slight increase in absorption above the *E*_g_, resulting in little to no loss in *J*_SC_ at elevated temperatures. From this analysis we can conclusively state that the change in *J*_SC_ is controlled by changes in the perovskite absorbance rather than interfacial charge extraction or recombination,^17^ in agreement with previous reports.^11,35^

**Figure 4:**
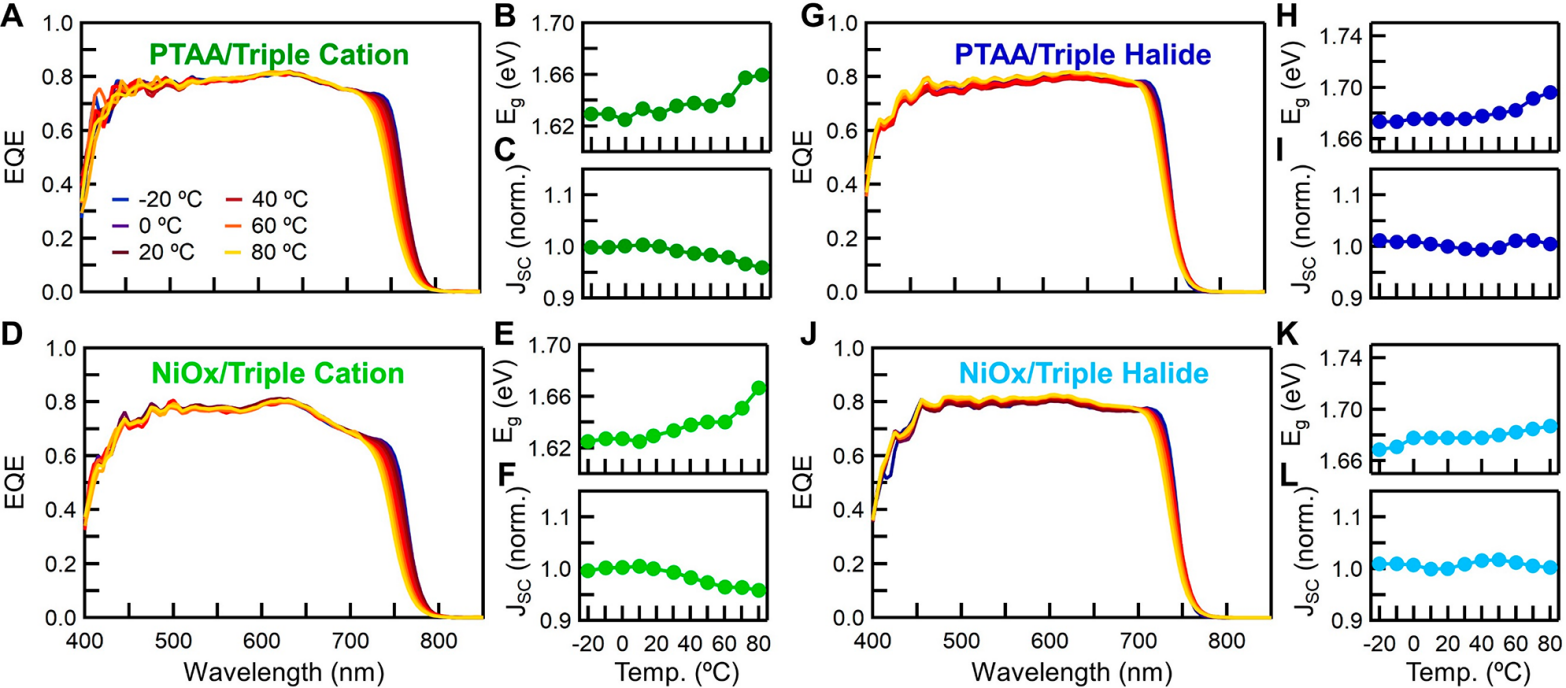
EQE curves, *E*_g_, as determined from the EQE spectra, and normalized integrated *J*_SC_ vs temperature for (A–C) PTAA/triple cation, (D–F) NiO_*x*_/triple cation, (G–I) PTAA/triple halide, and (J–L) NiO_*x*_/triple halide, respectively.

Next, we study the device specific changes in *V*_OC_ with temperature. In traditional PV technologies, such as Si or GaAs, the change in *V*_OC_ with temperature primarily drives the *T*_PCE_. Fundamentally, as temperature increases, wider band gap PV devices are less sensitive to changes in *V*_OC_ in part because the *relative* voltage loss is smaller than that of a narrower band gap PV device. Additionally wider band gap PV devices have smaller currents than narrower band gap PV devices; therefore, an equal finite change in voltage for both PV devices results in a smaller power loss in the wider band gap PV devices than the narrower band gap PV devices. Moreover, there are other critical factors that will also affect the temperature-dependent *V*_OC_ changes. To understand the general changes in *V*_OC_, we systematically go through the diode equations (eqs 2–4):
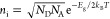
2
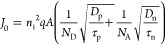
3
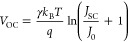
4where *N*_D_ and *N*_A_ are donor and acceptor density of states, respectively, *D*_p_ and *D*_n_ are hole and electron diffusion coefficients, respectively, *τ*_p_ and *τ*_n_ are hole and electron carrier lifetimes, respectively, *q* is elementary charge, *A* is area, γ is the ideality factor, and *k*_B_ is Boltzmann’s constant. First, an increase in temperature (*T*) increases the intrinsic carrier concentration (*n*_i_; eq 2), which increases the dark saturation current (*J*_0_; eq 3), causing a decrease in the *V*_OC_ (eq 4). Second, an increased thermal broadening, as described by the Fermi–Dirac distribution function (Table S3), will decrease *V*_OC_. Third, Si and GaAs *E*_g_ narrows at elevated temperatures as a result of the combined effects of lattice expansion and an increase in phonon density,^56^ leading to a further increase in *n*_i_ (eq 2), increasing *J*_0_ (eq 3) and further decreasing *V*_OC_ (eq 4).^57^ As expected, the fundamental increase in phonon density at elevated temperatures is also present for perovskites as evidenced by the positive correlation between Urbach energy (*E*_U_) and *T* (Figure 5). The increased *E*_U_ signifies an increase in the dynamic disorder and an enhancement of electron–phonon interactions, increasing *n*_i_ just as for Si and GaAs.^21,58,59^ However, the *E*_g_ shift for perovskites is *opposite* that for Si and GaAs and therefore should suppress the increase in *n*_i_ as opposed to exacerbating it.

**Figure 5:**
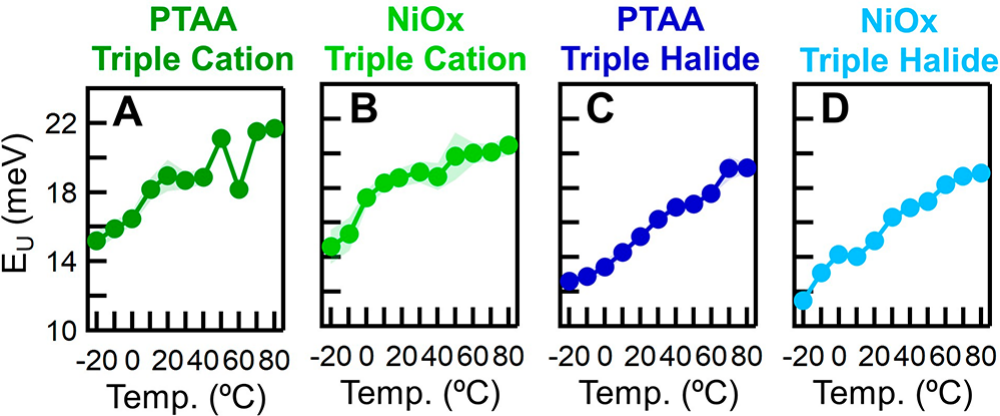
Representative device Urbach energy (*E*_U_) (A) PTAA/triple cation, (B) NiO_*x*_/triple cation, (C) PTAA/triple halide, and (D) NiO_*x*_/triple halide, respectively. Triple cation refers to FA_0.79_MA_0.16_Cs_0.05_Pb(I_0.83_,Br_0.17_)_3_, and triple halide refers to FA_0.75_Cs_0.22_MA_0.03_Pb(I_0.82_Br_0.15_Cl_0.03_)_3_.

To further understand these conclusions, we turn to drift-diffusion modeling which can elucidate the effect of different semiconductor properties on device *JV* characteristics. The model solves a system of time-dependent differential equations that dictate generation, movement, and recombination of charge carriers and has recently been shown to be accurate for perovskite photovoltaics.^41,60^ We first modeled the temperature-dependent performance of Si and GaAs (Figure 6), and their simulated *T*_PCE_ values match well to experimentally reported values, validating our model.^61^ Next, a triple cation device with ideal contacts was modeled (Figure 6). As with perovskites, GaAs also has a wider band gap than Si and thus both perovskites and GaAs will inherently have a lower *T*_PCE_ than Si. Fundamentally, because intrinsic carrier concentration is anticorrelated to band gap and the dark saturation current is proportional to the square of intrinsic carrier concentration (eq 3), the dark saturation current is also anticorrelated to the band gap. As temperature increases, the intrinsic carrier concentration increases (eq 2), and therefore the dark saturation current will also increase (eq 3). This relationship implies that when comparing two semiconductors at the same temperature, the wider band gap material will always have a lower dark saturation current. (Figure 6A−C, black lines) The perovskite compositions investigated in this study all have a wider band gap than GaAs and Si and thus will have lower limits on *T*_PCE_. Nevertheless, finer effects such as the *E*_g_ shift with temperature (as we show here with perovskites) will play an active role in determining the ultimate *T*_PCE_ (Figure 6A−C, red lines)

**Figure 6:**
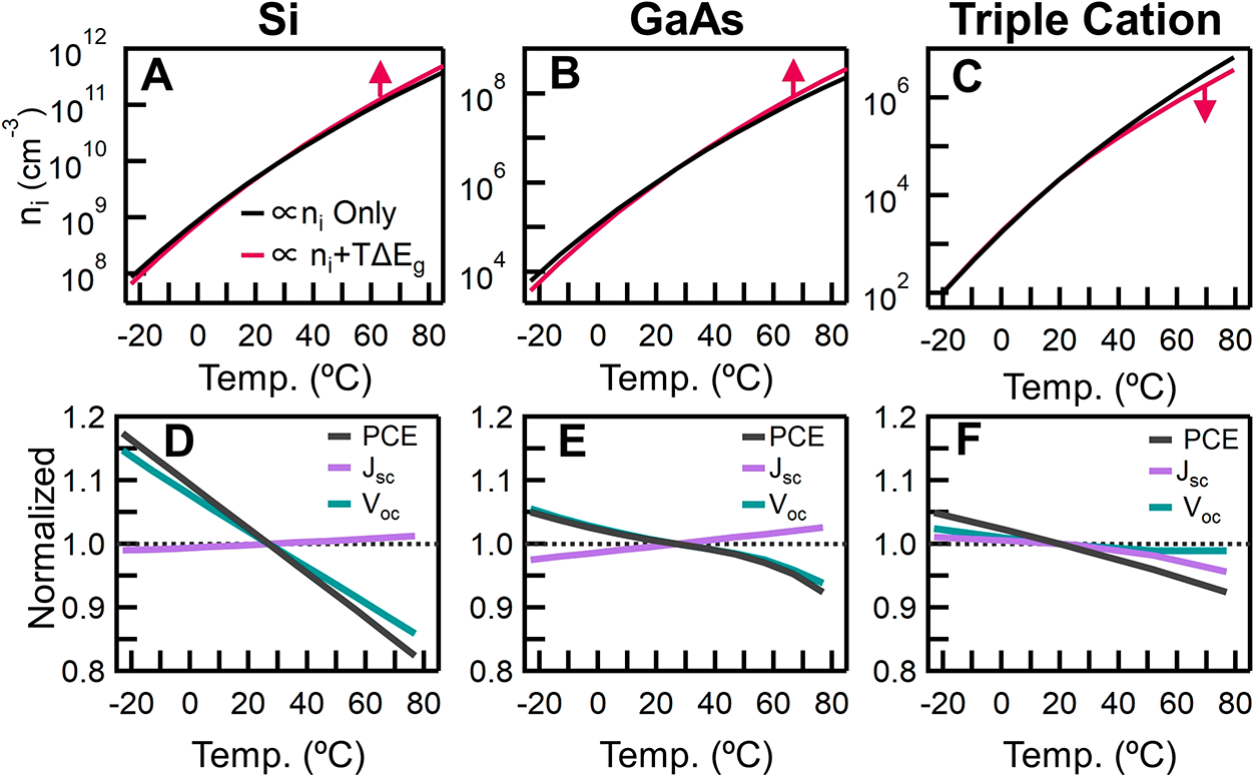
Drift-diffusion modeling of Si, GaAs, and triple cation perovskite solar cells. The modeled change in the intrinsic carrier concentration (*n*_i_) as a function of temperature and the change in *E*_g_ for (A) Si, (B) GaAs, and (C) triple cation perovskites. The modeled changes in PCE, *J*_SC_, and *V*_OC_ in (D) Si, (E) GaAs, and (F) triple cation perovskite as a function of temperature. The model assumes ideal contacts and both radiative and nonradiative bulk recombination processes to approximate the *JV* characteristics. Accounting for the shift in *E*_g_ results in the dampening of the intrinsic carrier concentration of the perovskite solar cells. This is further reflected in the minimal change in *V*_OC_ as a function of temperature and a consequent low *T*_PCE_, especially compared to silicon.

The band gap of perovskites increases with temperature instead of decreasing as it does in Si and GaAs. To determine the impact of the changes in band gap with temperature on device performance, we performed the simulations using the experimentally measured band gaps. The red curves in Figure 6A−C show the results with the varying band gap, while the black curves show the results when the band gap is considered to be constant with temperature. The model confirms our hypothesis, that the temperature-dependent blue shift in the *E*_g_ for perovskites will positively influence the *T*_PCE_. Figure 6C shows that the increase in the rate of intrinsic carrier concentration with temperature *suppresses* for perovskites when incorporating the changes in the *E*_g_ with temperature (red line), yielding the trends in *J*_SC_, *V*_OC_, and PCE, as shown in Figure 6F. Consequently, we probe deeper into the effects of the bulk and interfacial properties for the triple cation perovskite (Figure 7) to understand the current limitations of the perovskite composition and device architecture. The model clearly shows that the change in the *E*_g_ affects *J*_SC_ more than recombination (Figure 7A), supporting our conclusion that the changes in the perovskite absorbance is driving the changes in *J*_SC_. However, the changes in *V*_OC_ are not well modeled (Figure S11A–C) when only the bulk perovskite properties are incorporated. This discrepancy suggests that the increase in the *E*_g_ with temperature is not exclusively driving the low-*V*_OC_ losses for perovskites as compared to Si.

**Figure 7:**
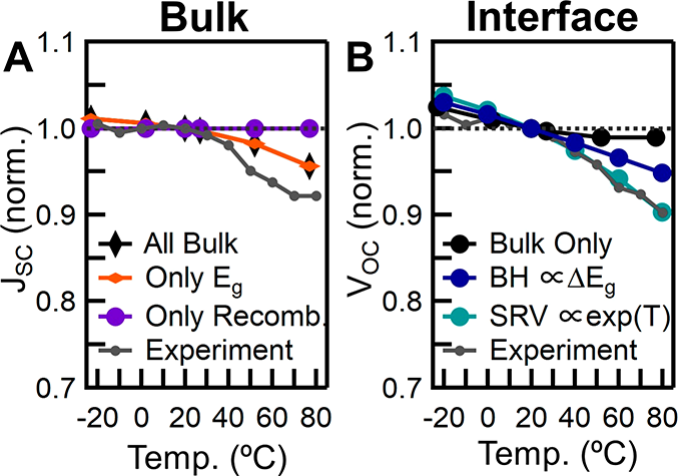
Drift-diffusion modeling of the temperature-dependent *J*_SC_ and *V*_OC_ for triple cation devices. (A) *J*_SC_ when only bulk perovskite properties are included (ideal contacts). (B) *V*_OC_ using non-ideal contacts separating out a change in barrier height (BH), proportionate to the change in *E*_g_, with a constant surface recombination velocity (SRV) and an exponential change in the surface recombination velocity with temperature, at a constant barrier height. Experimental data are the averages from PTAA/triple cation.

To better understand the observed *V*_OC_ losses, the effects of non-ideal contacts were modeled (Figure 7B). First, the influence of barrier height is studied at a constant surface recombination velocity (SRV). A change in the energetic barrier height proportional to the change in the *E*_g_ vs temperature accurately predicts the changes in *V*_OC_ seen experimentally. However, one must incorporate the fact that the ETL and HTL will exhibit changes in *E*_g_ and therefore also influence energetic barrier height. The *V*_OC_ is more susceptible to changes in barrier height than the *J*_SC_ as the *V*_OC_ is determined by the difference between the highest energy valence band and the lowest conduction band of the complete device, whereas significant band bending may allow charge carriers to be extracted even in the presence of an energetic barrier at the interface.

Next, the influence of the SRV is studied at a constant barrier height. An exponential temperature dependence has been previously proposed for monomolecular recombination in MAPbI_3_,^22^ and this relationship best matches our experimental data, suggesting that the temperature-dependent SRV is driven by specific recombination type(s) rather than a simple change in charge carrier thermal velocity.

Analysis of our data and literature reports shows that the improvement in *T*_PCE_ is not correlated to the starting room-temperature PCE nor the *E*_g_ (Figure S12). Combining the results from the drift-diffusion model and *JV* data, we find that the *T*_PCE_ is currently limited by the temperature-dependent *change* in interfacial energetic alignment and SRV, which are specific to a given device architecture, and not exclusively the shift in *E*_g_ and ensuing compromise between changes in *V*_OC_ and *J*_SC_. Therefore, to completely utilize the advantageous intrinsic properties of perovskites, a wider and a blue shifting *E*_g_ at increasing temperatures, both interfaces must be further optimized to ensure there is minimal SRV and optimal band alignment.

To determine the real-world implications of the temperature-dependent performance, we calculate the energy yield in 6 different U.S. cities with variable annual solar irradiances and average temperature (Figure 8). The effect on irradiance can vary between different device architectures, as each architecture will consist of different transport materials and perovskite compositions and therefore will have differing interface energetics and parasitic absorption losses. We focus on the best *T*_PCE_ perovskite device architecture, NiO_*x*_/triple cation, measure the normalized efficiency under variable illumination and temperature (Figure S13) and compare that to literature values for Si.^9^ We simulate the effects of the *T*_PCE_ and light intensity on the solar cells in the total energy yield. We take the current world record module efficiencies of a perovskite module (17.9%) and multicrystalline Si (20.4%),^7^ and apply our measured *T*_PCE_ and performance under variable illumination intensity. We assume a two-axis tracking system (direct illumination), and at this time ignore stability concerns of perovskite solar cells and calculate energy yield using standard considerations. To accurately determine the module temperature, we parsed the ambient temperature, solar irradiation intensity, device power output (data shown in Figure S13) vs temperature and intensity, and wind speed data of each location,^62^ into our model, and we determined the steady state module temperature every 30 min. It must be noted that ideally, the energy yield model would use the standard nominal module operating temperature (NMOT) metric, which includes the effect of the encapsulation and packaging materials on the operating temperature of the module. However, Jost et al. have shown that perovskites and silicon have a similar NOMTs, 43 and 44 °C, respectively,^11^ when using the current state-of-the-art perovskite encapsulation methods and thus we assume that NOMT of both the perovskite and Si solar cells are the same. Using the estimated module temperature and the measured efficiency at each temperature, the energy yield was calculated. The energy yield determination presented here takes into account both the fluctuations in PCE from intensity and PCE fluctuations from operating temperature using the determined temperature coefficients of performance.^11−13,63^ 17.9% PCE, NiO_*x*_/triple cation modules would generate within 95–97% of the power of that would be generated from 20.4% Si modules in all cities. Although it may be expected that Si would heavily outperform perovskites in cities with a cooler average temperature, these cities also have lower average irradiances (i.e., more cloud coverage) and perovskites have been previously shown to outperform Si in low light intensity environments (Figure S13).^64,65^ Additionally, to their benefit, perovskites have a wider *E*_g_ and thus are less sensitive than silicon to high atmospheric water vapor content found in warmer climates, since some water vapor absorption bands are not in their response range.^66^ Therefore, including the effect of atmospheric water vapor into the model, they will hinder the energy yield of Si; however, this would require spectral-dependent weather data for multiple cities, which is not readily available. This combination of higher relative efficiencies at low illumination and elevated temperatures is key for high energy yield. An in-depth investigation by Aydin et al. has shown that current state-of-the-art perovskite solar cells suffer from delamination of contacts after a few days of outdoor testing.^26^ Therefore, the energy yield calculations show that with significant further improvements to perovskite material, overall device stability, and innovative packaging they can challenge the well-established PV technologies.

**Figure 8:**
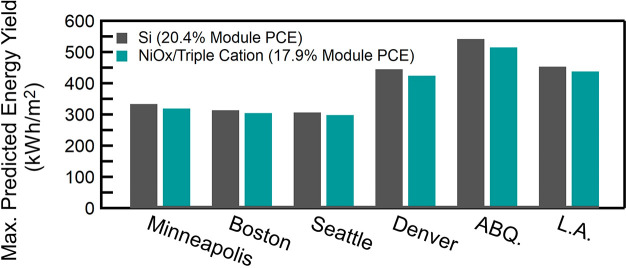
Calculated energy yield for a 17.9% NiO_*x*_/triple cation and a 20.4% Si solar cell for Minneapolis, MN; Boston, MA; Seattle, WA; Denver, CO; Albuquerque (ABQ.), NM; and Los Angeles (L.A.) CA.

Through a detailed comparison in the PV performance vs temperature between PTAA/triple cation, NiO_*x*_/triple cation, PTAA/triple halide, and NiO_*x*_/triple halide, we have measured a *T*_PCE_ of −0.08 rel %/°C and have identified key properties that affect the *T*_PCE_ of perovskite PV. First, the change in PCE vs temperature of any perovskite PV will be influenced by a combined change in the *J*_SC_, and *V*_OC_, unlike Si which is predominantly affected by the *V*_OC_. Our analysis shows that in general the perovskites used for PV have a wider band gap than Si and GaAs, and thus will fundamentally have a lower limit in *T*_PCE_. We also highlight the importance of the rate in *E*_g_ shift, which affects both the *J*_SC_ and *V*_OC_, either directly or indirectly by affecting *n*_i_. The device specific interfaces, either through changes in the surface recombination velocity and/or barrier height, also play a significant role in temperature-dependent changes in *V*_OC_. However, neither the room-temperature *E*_g_ nor PCE accurately predicts the resulting *T*_PCE_; thus, the specific temperature-dependent properties of both the contacts and perovskite absorber must be considered when designing and deciding which device architecture will be employed in the field.

The results and analysis presented in this study conclusively show that perovskite-based photovoltaics have advantages in key intrinsic semiconductor properties compared to existing technologies and by improving the stability of them will make them a highly competitive commercial PV technology.

## Supporting Information Available

Details on the experimental procedures, supporting figures and the parameters used for the drift-diffusion modeling and energy yield calculations (PDF)
