# Supplementary material for: Temperature Coefficients of Perovskite Photovoltaics for Energy Yield Calculations
Source: ACS Energy Lett. 2021 May 7;6(5):2038–47. doi: 10.1021/acsenergylett.1c00748 (PMC10157636; doi:10.1021/acsenergylett.1c00748)
Supplement: Supplementary file 1 — nz1c00748_si_001.pdf [file nz1c00748_si_001.pdf]

Temperature Coefficients of Perovskite Photovoltaics for Energy Yield Calculations

Taylor Moot[1†], Jay B. Patel[1,2†], Gabriel McAndrews[1,3], Eli J. Wolf[1,2,4], Daniel Morales[1,3], Isaac E. Gould[1,3], Bryan A. Rosales[1], Caleb C. Boyd[1,2,5], Lance M. Wheeler[1], Philip A. Parilla[1], Steven W. Johnston[1], Laura T. Schelhas[1], Michael D. McGehee[1,2,3*], Joseph M. Luther[1*]

[1]National Renewable Energy Laboratory, Golden CO, 80401, USA
[2]Department of Chemical and Biological Engineering, University of Colorado, Boulder, CO 80309, USA
[3]Materials Science and Engineering, University of Colorado, Boulder, CO 80309, USA
[4]Department of Applied Physics, Stanford University, Stanford CA, 94305, USA
[5]Department of Materials Science and Engineering, Stanford University, Stanford, CA 94305, USA

[†]Equal Contribution
[*]Corresponding Author: Joey.Luther@NREL.gov, Michael.McGehee@Colorado.edu

Supporting Information

Experimental Procedures

Chemicals. All chemicals were bought from Sigma Aldrich and used without further purification unless stated otherwise. Methylammonium bromide and formamidinium iodide (GreatCell Solar Materials), PbI_2 (TCI America), Poly(triaryl amine) (PTAA, Solaris Chem), poly(9,9-bis(3'-(N,N-dimethyl)-N-ethylammoinium-propyl-2,7-fluorene)-alt-2,7- (9,9-dioctylfluorene))dibromide (PFN-Br, 1-Material), NiO target (99.9% stoichiometric, Kurt J. Lesker) were all obtained elsewhere as indicated.

Metal Halide Perovskite Fabrication. $FA_{0.79}MA_{0.16}Cs_{0.05}Pb(I_{0.83},Br_{0.17})_3$ (Triple Cation) was fabricated using previously reported methods.[1] Briefly, a 1.26 M solution was made in 4:1 v/v DMF:DMSO. The perovskite solution was deposited and spun at 5000 r.p.m. for 35 s, and 300 μL of ethyl acetate was deposited in a continuous stream with 25 s left in the spin procedure. The film was then annealed at 100 °C for 60 min. $FA_{0.75}Cs_{0.22}MA_{0.03}Pb(I_{0.82}Br_{0.15}Cl_{0.03})_3$ (Triple Halide) was fabricated using previously reported methods.[2] Briefly, a 1.4 M solution was made in 3:1 v/v DMF:DMSO. The perovskite solution was deposited and spun at 5000 r.p.m for 50 s, and 200 μL of methyl acetate was deposited in a continuous stream with 22 s left in the spin procedure. The film was then annealed at 100 °C for 30 min. For perovskites deposited on NiO_x, 6-8 mol% excess FAI was added to the solution in accordance with previous reports.[3]

Device Fabrication
All substrates (glass or ITO for solar cells) were cleaned by sonicating in isopropanol for 15 min. followed by UV-ozone immediately before deposition. For any MHP characterization the perovskite was deposited directly onto cleaned glass. For PTAA based devices, a 1.5 mg/mL solution in toluene was spun at 6000 r.p.m. for 30 s then the film was annealed at 100 °C for 10 min. Subsequently, a 0.5 mg/mL PFN-Br in methanol wetting layer was deposited dynamically at 5000 r.p.m. and spun for 30 s. For NiO_x based devices, the cleaned substrates were loaded into a Denton sputter tool and pumped down to <5 x 10^{-6} torr. After a 15 min. pre-sputter target cleaning, NiOx was deposited via RF magnetron sputtering at 60W and 16.5 mTorr in pure Ar at room temperature. The perovskite layer was then deposited as discussed above. All devices were completed by thermal evaporation of LiF (1 nm, 0.1 A/s), C_{60} (30 nm, 0.2 A/s for 5nmn then 0.5 A/s for 25 nm), BCP (6nm 0.2 A/s) and Ag (100 nm, 0.5 A/s for 5 nm, then 2 A/s for 95 nm) in an Angstrom evaporator with a base pressure of <8 x 10^{-7} torr.

UV-visible absorption spectroscopy. and E_g Calculation
Absorbance measurements were performed in a home-built system using an optical holder sealed under N_2. The sample was illuminated with a collimated 550W xenon arc lamp and measurements were collected with an OceanOptics Maya 2000 Pro High Sensitivity Spectrometer. The temperature was controlled using a Huber Pilot ONE thermoregulator. Bandgaps were calculated using a Tauc plot and fitting to the large linear component (ignoring the tail).

X-ray diffraction and analysis.
The lattice-constant was determined with in-situ x-ray diffraction (XRD) using a Bruker D8 Discover with a Vantec area detector (sample-to-detector distance of 15 cm) and an Anton Paar DHS 900 hot stage. The sample was placed on the hot stage with spring clips and illuminated with x-rays from a copper target (40 kV, 35 mA) using a Göebel mirror (parallel optics) and

1mm circular collimator. The hot stage reached the indicated temperature (and identical to heater setpoint), before the 5-minute XRD data frames centered at 30° 2-theta were acquired in an air environment. It is expected that the actual surface temperature of the sample is somewhat less than the setpoint temperature. The two-dimensional data from the detector was integrated along the chi axis in steps of 0.02° of 2-theta to produce the intensity versus 2-theta data used in the lattice determination with a Pawley fit using the pm-3m space group. Fitting was performed using the TOPAS-Academic software package

Solar Cell JV Characterization. Room temperature JV measurements (Figure S2) were performed in a nitrogen box using a Newport Oriel 94043A Sol3A Class AAA solar simulator that was calibrated to 1-sun intensity each time before use with a NREL calibrated Si photodiode and a KG2 filter. Devices were masked using a 0.058 cm^2 shadow mask and a scan rate of 0.47 V/s was used. Temperature dependent JV measurements were performed in N_2 on a custom-built ScienceTech Class AAA solar simulator that was calibrated to 0.85-sun intensity each time before use with a NREL calibrated GaAs photodiode. The actual intensity of the solar simulator was 1 sun, however due to reflection losses from the cryostat windows there was a drop in intensity to 0.85 suns directly onto the solar cell. Devices were unmasked and have an area of 0.11 cm^2. The temperature was controlled using a Huber Pilot ONE thermoregulator and the solar cells were equilibrated at each temperature for ~5 minutes before measuring performance. Temperature and intensity dependent JV measurements were performed under vacuum using Newport Oriel model 67005 arc lamp housing with xenon lamp type UXL-150SO solar simulator that was calibrated to 1-sun intensity each time before use with a NREL calibrated Si photodiode. A p-i-d controlled temperature controller was used to modulate the temperature and neutral density optical filters were used at each temperature. The steady state power output was measured in vacuum using an Oriel 91160 solar simulator. Initially the voltage was set at 0.7V and MPPT tracking was carried out for 60 seconds. All measurements were first taken at room temperature (20 ^oC), then from the coldest (-20 ^oC) to the highest temperature (80 ^oC) in 10 ^oC or 20 ^oC steps and finally back at room temperature (20 ^oC).

To ensure that the effect of illumination on the temperature of the perovskite solar cell was accounted for, we placed a platinum thermocouple on the perovskite solar cell and measured the temperature increase over time as a solar simulator with 1 sun intensity was irradiated upon the solar cell.

The 1 sun illumination onto the solar cell induced a negligible increase in the temperature when measuring a JV scan, which in our experiment lasted 3.5s as shown below (~0.2 K). Therefore, we used the initial temperature of the solar cell for the reported temperature of each JV parameter in the manuscript. However, carrying out a steady state power output measurement for 60 s resulted in a ~3K increase in the solar cell temperature, thus the final temperature was used as the reported temperature.

Initial Temp (t=0)	Final temp (t=67.5)	Difference (K)	K/s	Temperature change per 3.5 s JV scan	Temperature change per 60 s MPPT
253.37	257.66	4.29	0.06	0.22	3.84
262.83	267.06	4.23	0.06	0.22	3.79
273.35	277.38	4.03	0.06	0.21	3.61
283.00	286.98	3.98	0.06	0.21	3.56
292.88	297.09	4.21	0.06	0.22	3.77
303.53	307.45	3.92	0.06	0.20	3.51
313.30	316.93	3.63	0.05	0.19	3.25
323.22	326.52	3.30	0.05	0.17	2.96
332.62	336.16	3.54	0.05	0.18	3.17
342.06	345.58	3.52	0.05	0.18	3.15
352.78	356.60	3.82	0.06	0.20	3.42

Solar Cell EQE Characterization. The MHP device was placed in a cold finger cryostat (Janis ST-100H) with a platinum thermocouple affixed onto the MHP device for reliable and accurate measurement of the temperature of the device. The temperature was controlled using a second sensor on the cold finger itself. The devices were cooled using liquid nitrogen. Measurements were taken from the coldest (-20 °C) to the highest temperature (80 °C). A Newport quartz tungsten halogen lamp was used as the light source and a monochromator (Acton Research SP150) was used to select the desired excitation wavelength. The light from the lamp had to pass through a quartz window of the cryostat, which reduced the intensity of light below 600nm, due to reflection losses, resulting in lower relative signal at lower wavelengths of light. In order to increase measurement sensitivity, the measurements were performed using a chopped signal (173Hz) to enable the use of lock-in amplifiers (SRS 830). A reference diode was used to ensure that any fluctuations in light intensity were accounted for in the duration of the experiments. The photocurrent spectra were then corrected using a NREL calibrated diode of known EQE, (Newport 818-SL). The Eg was extracted from the EQE spectra by calculating the derivative of the spectra and finding the inflection point. The Urbach energy was extracted by interpolating the data of EQE spectra near the band edge. A MATLAB non-linear regression was used to fit the data. The robust fitting was performed using a bisquared (Tukey's biweight) weighting function and a 95% confidence interval was extracted for the Urbach energy using the residuals and Jacobian of the fit.[4]

Temperature Coefficient calculation. The T_{PCE} was calculated as [PCE(HT)-1]/[T(HT)-T(RT)], where PCE(HT) is the normalized efficiency with respect to the room temperature PCE (~25°C) and T(HT) is the temperature at the high temperature and the RT is the room temperature (~25°C)

R_{sh} +R_s Calculation. J_0 was measured by taking the y-intercept of a dark JV curve. The dark JV curves were taken with the solar simulator turned off, however there was still some background light from the laboratory and the equipment (displays of instrumentation). R_{SH} and R_S were calculated from the slope of the JV curve (dark and light) at the y-intercept and the x-intercept, respectively.

Drift-Diffusion Modeling. Briefly, the model uses a series of coupled differential equations relating to species movement due to concentration gradients, diffusion, as well as charged species response

to local electric field drift. The details of the model and its equation can be found in the previous report.[5] Simulating temperature dependence requires the use of several parameters which can be found in Table S1 and S2. Temperature dependent trends were applied to parameters from solid state physics definitions, perovskite solar cell literature, or from experimentation discussed in this work.

Energy Yield Modeling. Weather data[6] and Si solar cell[7] temperature and intensity dependent power were obtained from previous reports. All energy yield calculations were performed using Python 3 and source code is available upon request. Experimental device data was linearly interpolated using scipy's interpolate grid data function to allow for interfacing with the weather data. In order to compare perovskite and silicon cells of different efficiencies, the power matrix was scaled so that the efficiency at 1 Sun and 20 ^oC matched the specified efficiency (20% for Si and 20% or 17.9% for perovskite). The weather data provides insolation, wind speed, and ambient temperature at 30-minute intervals for an entire year. Using the insolation (I), wind speed (w), and ambient temperature ($T_{ambient}$) for a given point in time, the cell temperature was calculated by solving the following equation:

$$0 = G(I, T_{cell}) + R(T_{cell}, T_{ambient}) + C(T_{cell}, T_{ambient}, w)$$

$G(I, T_{cell})$ is the amount of heat generated by the light from the sun on the panel, $R(T_{cell}, T_{ambient})$ is the amount of heat radiated by the panel assuming black body radiation, and $C(T_{cell}, T_{ambient}, w)$ is the amount of heat transferred between the panel and the ambient environment through convection. Conduction between the mounting hardware and the panel was assumed to be zero. The equations used to model the amount of convection was the same as previously reported.[8]

Once the panel temperature is determined, the insolation, from the weather data, and the panel temperature are used to find the power extracted by the cell using the power matrix. This process is repeated for every time chunk throughout the whole year. The power density is then summed to calculate the energy density for a full year. Dividing the annual energy density extracted by the panel with the total energy density incident on the panel, a weighted efficiency can be extracted. Due to the limited availability of the spectral databases for locations with varying climates and irradiance levels, the energy yield was calculated using the power generated from a device exposed to a standard AM 1.5 solar spectrum.

Supplemental Figures

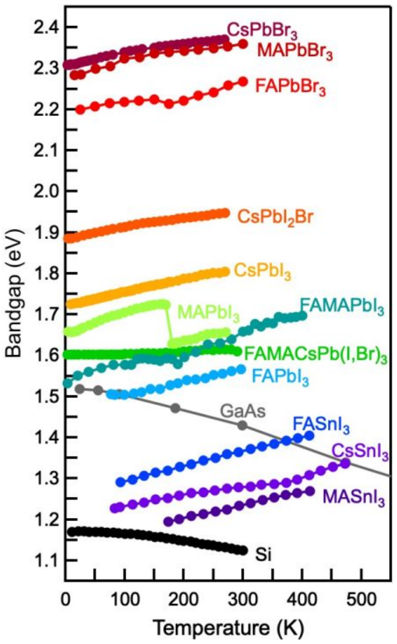

Figure S1: Change in E_g versus temperature from literature reports for $CsPbBr_3$,[9] $MAPbBr_3$,[10] $FAPbBr_3$,[10] $CsPbI_2Br$,[10] $CsPbI_3$,[10] $MAPbI_3$,[11] $FAMAPbI_3$,[12] $FAMACsPb(I,Br)_3$,[13] $FAPbI_3$,[14] $FASnI_3$,[15] $CsSnI_3$[15] and $MASnI_3$[15] compared to GaAs[16] and Si.[16]

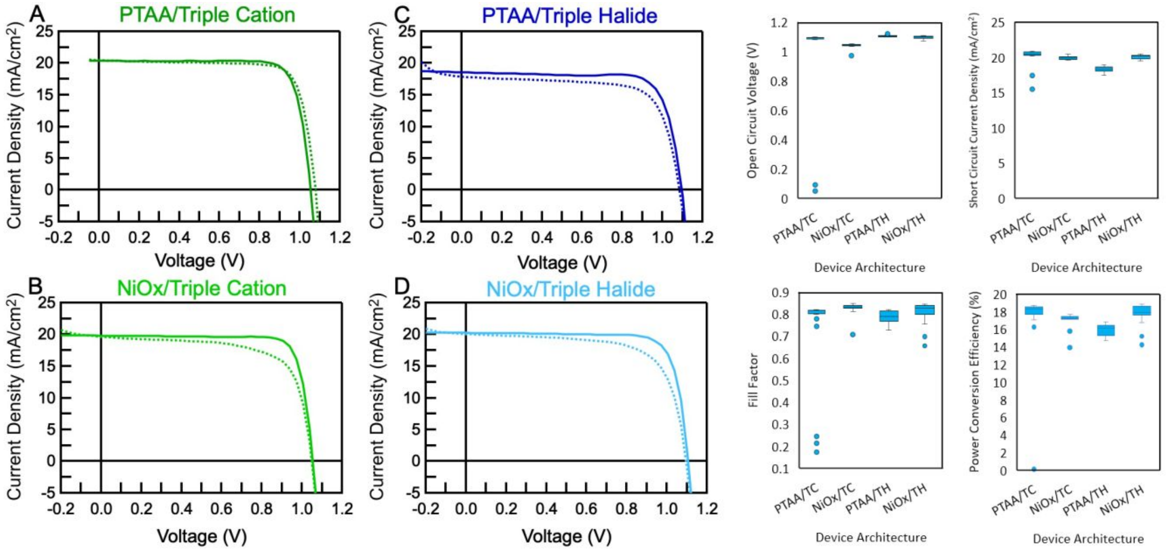

Figure S2: Representative JV curves (forward: dashed, reverse: solid) tested using AM1.5G 1-sun illumination from each batch for **A**) PTAA/Triple Cation (18.1% PCE), **B**) NiO_x/Triple Cation (17.2% PCE), **C**) PTAA/Triple Halide (15.9% PCE), and **D**) NiO_x/Triple Halide (17.9% PCE). The boxplots show distribution of the JV metrics of all devices used in this study.

Table S1: Normalized reverse curve PCE average (avg) ± standard deviation (stdev) compared to stabilized power output (SPO) for each device architecture and temperature. The device temperature changed by ~ 4 °C during the SPO measurement, explaining some of the slight deviations from the reverse curve PCE.

Temp (°C)	PTAA/Triple Cation Avg ± Stdev (norm.)	PTAA/Triple Cation SPO (norm.)	NiO_x/Triple Cation Avg ± Stdev (norm.)	NiO_x/Triple Cation SPO (norm.)	PTAA/Triple Halide Avg ± Stdev (norm.)	PTAA/Triple Halide SPO (norm.)	NiO_x/Triple Halide Avg ± Stdev (norm.)	NiO_x/Triple Halide SPO (norm.)
-20	1.01 ± 0.003	1.02	0.66 ± 0.099	0.52	0.96 ± 0.105	1.24	0.85	0.81
-10	0.99 ± 0.000	1.05	0.70 ± 0.048	0.72	1.03 ± 0.030	1.20	-	1.02
0	1.00 ± 0.013	1.04	0.86 ± 0.058	0.91	0.98 ± 0.074	1.12	0.94	1.11
10	1.00 ± 0.000	1.03	0.93 ± 0.012	1.00	1.02 ± 0.018	1.06	-	1.06
20	1.00 ± 0.000	1.00	1.00 ± 0.000	1.00	1.00 ± 0.000	1.00	1.00	1.00
30	0.98 ± 0.000	0.97	1.05 ± 0.006	0.94	0.98 ± 0.012	0.92	-	0.91
40	0.95 ± 0.011	0.93	1.00 ± 0.113	0.90	0.98 ± 0.051	0.89	0.88	0.89
50	0.90 ± 0.000	0.88	1.05 ± 0.031	0.88	0.95 ± 0.015	0.88	-	0.91
60	0.86 ± 0.025	0.84	0.98 ± 0.073	0.86	0.98 ± 0.119	0.87	0.90	0.91
70	0.82 ± 0.000	0.80	0.99 ± 0.030	0.88	0.94 ± 0.023	0.87	-	1.01
80	0.79 ± 0.035	0.75	0.93 ± 0.017	0.86	0.85 ± 0.092	0.86	0.86	1.06

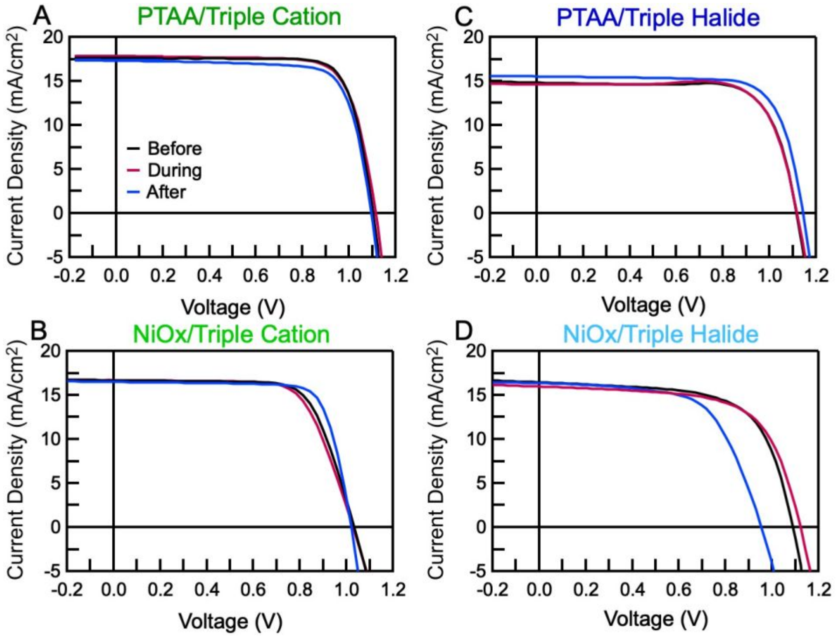

Figure S3: Reverse JV curves tested before (black), during (pink) and after (blue) thermal cycling for **A**) PTAA/Triple Cation, **B**) NiO_x/Triple Cation, **C**) PTAA/Triple Halide and **D**) NiO_x/Triple Halide.

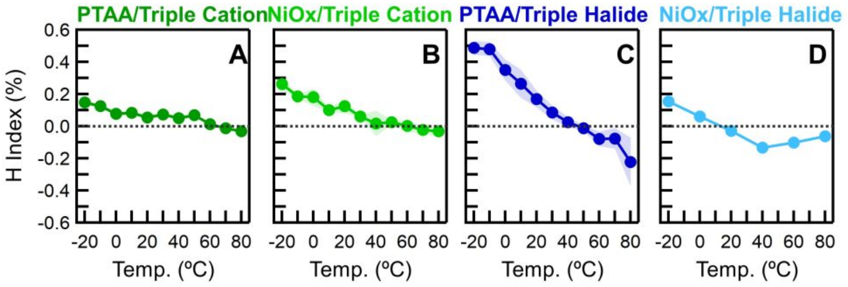

Figure S4: Average hysteresis index and standard deviation (shaded) as a function of temperature for **A**) PTAA/Triple Cation, **B**) NiO_x/Triple Cation, **C**) PTAA/Triple Halide and **D**) NiO_x/Triple Halide.

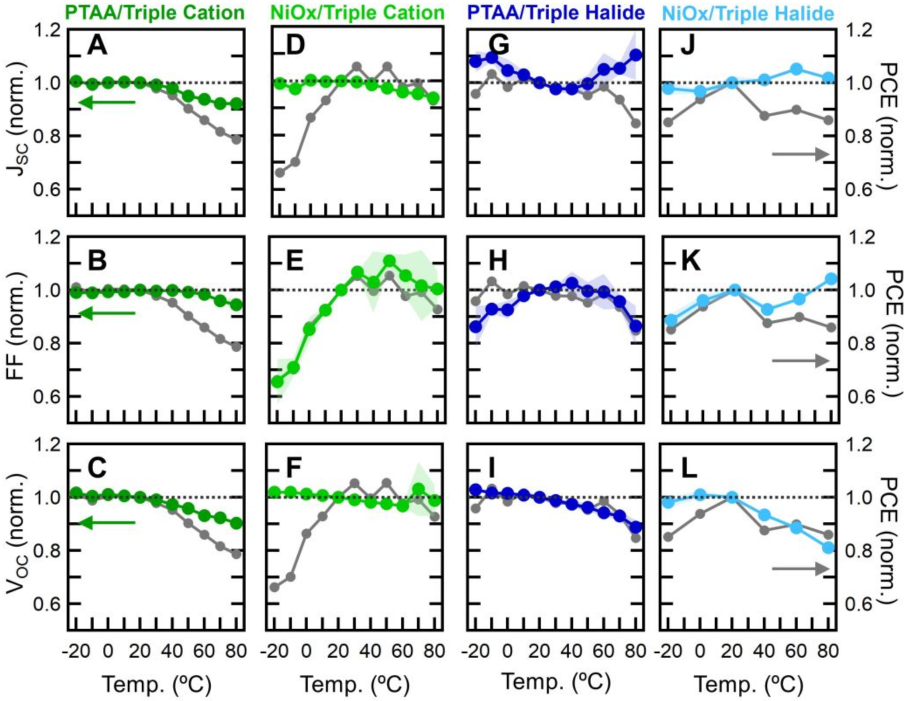

Figure S5a: Normalized J_{SC} (top row), FF (middle row) and V_{OC} (bottom row) as a function of temperature for **A, B, C**) PTAA/Triple Cation (dark green), **D, E, F**) NiO_x/Triple Cation (light green), **G, H, I**) PTAA/Triple Halide (dark blue) and **J, K, L**) NiO_x/Triple Halide (light blue), respectively. Grey trace is the normalized PCE (right axis).

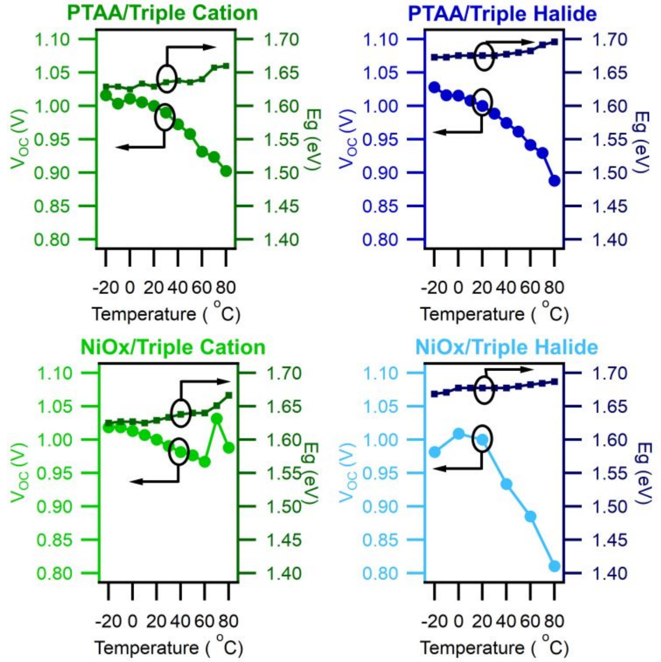

Figure S5b: The temperature dependence of the V_{oc} and the E_g of the various device architectures.

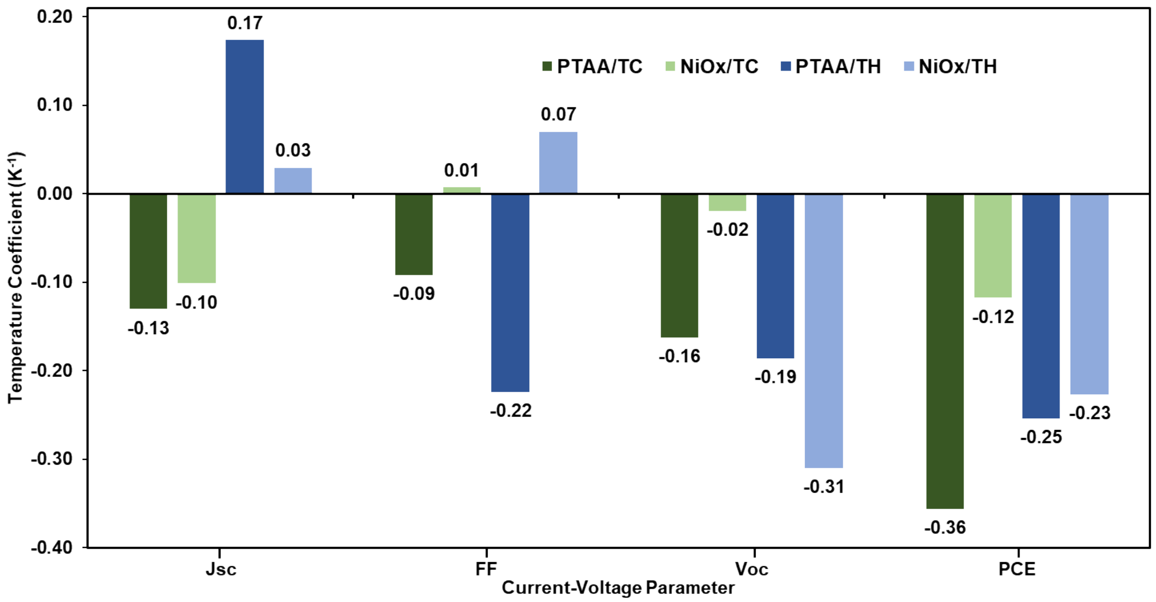

Figure S6. The average temperature coefficients of all the device types used, calculated from the averaged data in Figure S5. The temperature coefficient is calculated from the difference in the parameters between 80 °C and 20 °C. Overall it is clear that the temperature coefficient of the combined *JV* parameters are superior for the NiOx/Triple Cation device type, which is reflective of the champion TPCE we measure.

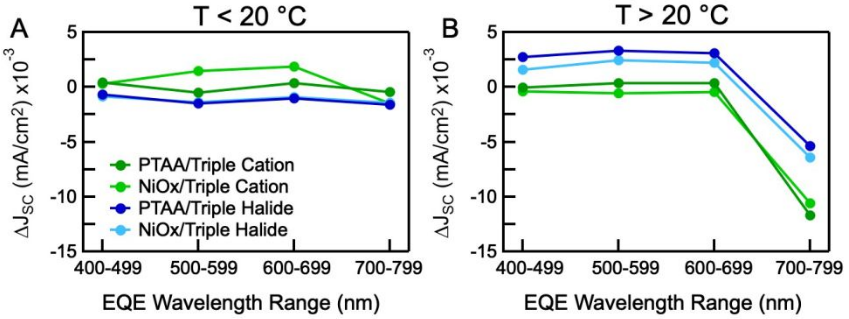

Figure S7: Relative change in J_{SC} as calculated from the EQE based on different spectral portions at **A**) low temperatures and **B**) elevated temperatures.

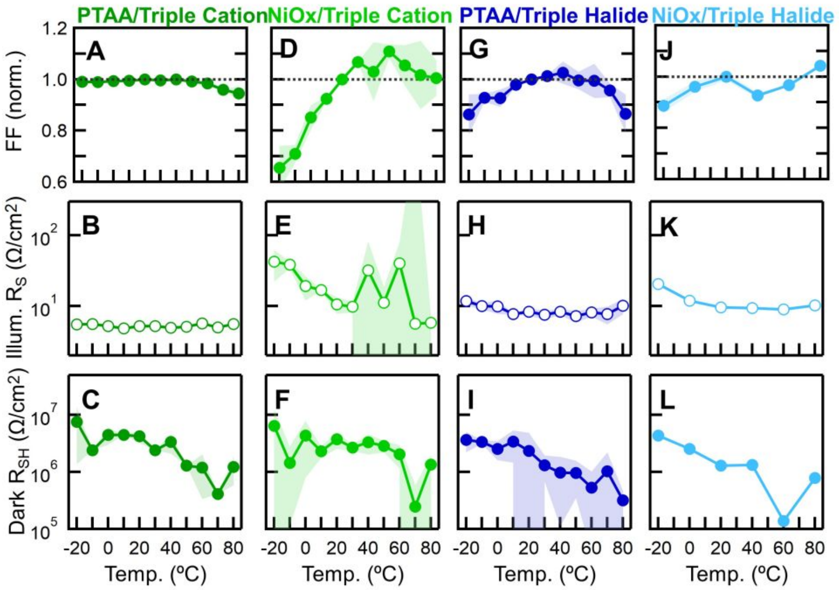

Figure S8: Average normalized FF with standard deviation (shaded) (top row) from the reverse *JV* sweep, illuminated R_S (middle row) and dark R_{SH} (bottom row) for **A, B, C**) PTAA/Triple Cation, **D, E, F**) NiO_x/Triple Cation, **G, H, I**) PTAA/Triple Halide and **J, K, L**) NiO_x/Triple Halide, respectively.

Note S1:All device architectures show a slight decrease in *FF* at elevated temperatures, but there are significant differences in the change in *FF* at low temperatures. For example, PTAA/Triple Cation exhibits no decrease in *FF* at low temperatures whereas NiO_x/Triple Cation decreases significantly down to 65%. In contrast to the trends in J_{SC}, the trends in *FF* do not appear to be tied to a specific absorber nor contact layer suggesting that the *FF* is controlled by the specific device architecture.

Generally, *FF* changes are driven series (R_S) or shunt resistance (R_{SH}). These can be temperature dependent due to changes in the resistance of the contact layers or changes in the available defect states in the perovskite, respectively.[17–19] Comparison of the R_S from illuminated devices shows a clear increase in R_S at low temperatures exclusively for NiO_x/Triple Cation devices. Notably, there is little to no change in the dark R_S at low temperatures (**Figure S8**) suggesting that the change in R_S is due to an interfacial charge transfer resistance. The charge transfer resistance is specific to the exact interface trap states and/or energetic alignment and is not driven here by any changes in the resistances of the contacts themselves. While some reports have suggested that there is an increase in R_S of the contacts at low temperatures,[7] more argue that this does not significantly affect the *FF*,[20,21] in agreement with our experimental data. As for the dark R_{SH}, there is a general decrease at elevated temperatures, suggesting the increased availability of trap states for all perovskite compositions, in agreement with previous reports.[19,21]

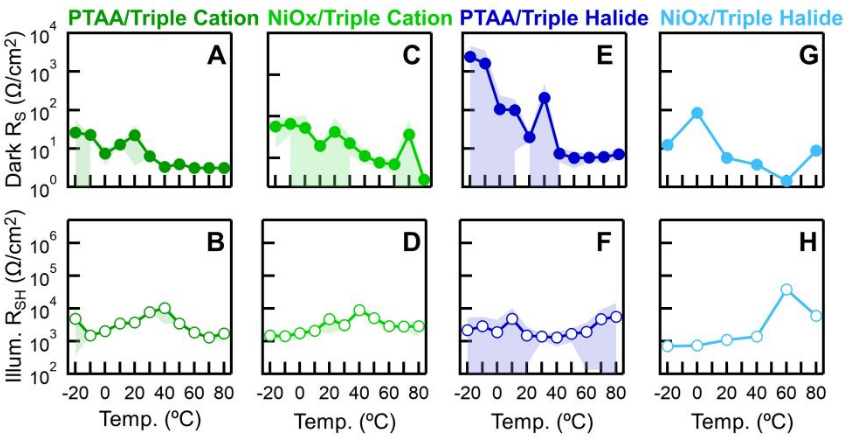

Figure S9: Average dark R_S (top row) and illuminated R_{SH} (bottom row) with standard deviation (shaded) for **A, B**) PTAA/Triple Cation, **C, D**) NiO_x/Triple Cation, **E, F**) PTAA/Triple Halide and **G, H**) NiO_x/Triple Halide, respectively.

Table S2: All parameters used in the drift-diffusion modeling of Si and GaAs, including bandgap (E_g), relative permittivity (ε_r), conduction band density of states (N_C), valence band density of states (N_V), electron mobility, hole mobility, B-coefficient and absorption coefficient (α) as a function of temperature(T).

Parameter	Silicon	GaAs	Ref.
$E_g\ eV$	$1.166-(0.473\text{x}10^{-3}T^2)/(T+636)$	$1.519-(0.541\text{x}10^{-3}T^2)/(T+204)$	22
ε_r	11.9	13.1	22
$N_c\ cm^{-3}$	$2.81\text{x}10^{19}(T/300)^{3/2}$	$4.35\text{x}10^{17}(T/300)^{3/2}$	22
$N_v\ cm^{-3}$	$1.83\text{x}10^{19}(T/300)^{3/2}$	$7.57\text{x}10^{18}(T/300)^{3/2}$	22
Electron mobility ($cm^2/(V{\cdot}s)$)	$7.27\text{x}10^8T^{-2.4}$	$2.16\text{x}10^6T^{-1}$	22
Hole mobility ($cm^2/(V{\cdot}s)$)	$8.5\text{x}10^7T^{-2.2}$	$3.1841\text{x}10^7T^{-2.1}$	22
B-coefficient	$3\text{x}10^{-10}\ (E_g/2)^2(300/T)^{3/2}$	$(3\text{x}10^{-10}\ (E_g/2)^2\ (300/T)^{3/2}$	23
α (cm^{-1})	$1\text{x}10^4$	$1\text{x}10^4$	24,25
β_p electron Capture Coefficient $\frac{cm^3}{s}$	~2E-18 cm^{-2} x Thermal Velocity	~2E-18 cm^{-2} x Thermal Velocity	26
β_n hole Capture Coefficient $\frac{cm^3}{s}$	~6E-15* exp(-0.066/kT) (cm^{-2})	~6E-15*exp(-0.066/kT)(cm^{-2})	26

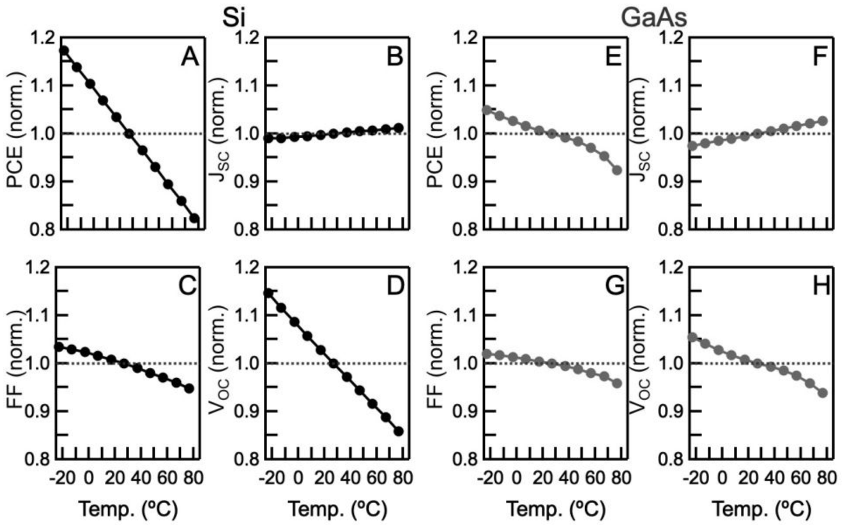

Literature	$\frac{d(Voc)}{dT}\left(\frac{mV}{K}\right)$	$\frac{d(Jsc)}{dT}\left(\frac{mA}{cm^2K}\right)$	$\frac{d(FF)}{dT}$	Ref.
GaAs	-2	2e-2	-6.55e-2 %/K	40
Silicon	-2.2	6e-4	-1.5e-1 / K	39

Simulated	$\frac{d(Voc)}{dT}\left(\frac{mV}{K}\right)$	$\frac{d(Jsc)}{dT}\left(\frac{mA}{cm^2K}\right)$	$\frac{d(FF)}{dT}$
GaAs	-1.86	1.04e-2	-6.47e-2 %/K
Silicon	-1.61	2e-4	-1.15e-1 / K

Figure S10: Drift-diffusion modeling of the trends in normalized PCE, J_{SC}, FF and V_{OC} versus temperature for **A, B, C, D**) Si and **E, F, G, H**) GaAs, respectively. The experimental and simulated temperature coefficients (T_C) for PCE, J_{SC}, FF and V_{OC} all match well as shown in the table.

Table S3: All parameters used in the drift-diffusion modeling of MHPs including relative permittivity (ε_r), electron injection barrier (b_n), hole injection barrier (b_p), equilibrium halide vacancy concentration (c_{eq}), photon flux (Φ), absorption coefficient (α), MHP thickness (L), thermal voltage (V_T), Triple Cation bandgap ($E_{G\text{-}TC}$), Triple Halide bandgap ($E_{G\text{-}TH}$), built in voltage (V_{bi}), conduction band density of states (N_C), valence band density of states (N_V), equilibrium electron concentration (n_0), equilibrium hole concentration (p_0), intrinsic carrier concentration (n_i), electron/hole mobility (μ), electron diffusion coefficient (D_e), hole diffusion coefficient (D_p), halide vacancy diffusion coefficient (D_v), bimolecular recombination coefficient (B), Shockley-Read-Hall trap density (N_{trap}), electron capture coefficient (β_n), hole capture coefficient (β_p), electron surface recombination velocity (S_e) and hole surface recombination velocity (S_p) as a function of bandgap (E_g) and temperature (T).

Parameter	T=300K Value	T Dependent Value	Ref.
ϵ_r	24	24	5
b_n	$0.3\ eV$	$0.3 + A\big(E_g(T) - E_g(300K)\big)$ A=1, 5 (proportionality coefficient)	5
b_p	$0.3\ eV$	$0.3 + A\big(E_g(T) - E_g(300K)\big)$ A=1, 5	5
c_{eq}	$1x10^{17}cm^{-3}$	$1x10^{17}cm^{-3}$	5

Φ	$4.546x10^{17} + (3.307x10^{16})E$[illegible]	From $E_g(T)$	
α	$2.5x10^{4}\ cm^{-1}$	$2.5x10^{4}\ cm^{-1}$	[27]
L	500 nm	500 nm	
V_T	$0.026\ eV$	$K_B T\ \ eV$	
E_{g-TC}	$1.649\ eV$	$(2.344x10^{-5})e^{2.178x10^{-2}T} + 1.63$[illegible]	Fig. S6A
E_{g-TH}	$1.7145\ eV$	$1.02 + (7.34x10^{-3})T - (2.62x1$[illegible]	Fig. S6A
V_{BI}	$E_g - b_n - b_p$	$E_g(\mathrm{T}) - b_n(\mathrm{T}) - b_p(T)$	
N_C	$3.0x10^{18}\ cm^{-3}$	$5.8x10^{14}T^{3/2}\ \ cm^{-3}$	[28]
N_V	$3.0x10^{18}\ cm^{-3}$	$5.8x10^{14}T^{3/2}\ \ cm^{-3}$	[28]
n_0	$2.92x10^{13}\ cm^{-3}$	$N_C\, e^{-b_n/K_BT}\ \ cm^{-3}$	
p_0	$2.92x10^{13}\ cm^{-3}$	$N_V\, e^{-b_p/K_BT}\ \ cm^{-3}$	
n_i	$1.3x10^{5}\ cm^{-3}$	$\sqrt{N_C N_V} e^{-E_g/K_BT} cm^{-3}$	
μ	$28.87\,\frac{cm^2}{Vs}$	$1.5x10^{5}\, T^{-1.5}\ \frac{cm^2}{Vs}$	[29]
D_e	$0.75\ \frac{cm^2}{s}$	$\mathrm{\mu} K_B T\ \ \frac{cm^2}{s}$	
D_p	$0.75\ \frac{cm^2}{s}$	$\mu K_B T\ \ \frac{cm^2}{s}$	
D_{vac}	$1x10^{-7}\frac{cm^2}{s}$	$1.5x10^{-2}\ e^{-0.3/K_BT}\ \ \frac{cm^2}{s}$	[29]
B	$2x10^{-10}\ \frac{cm^3}{s}$	$5.0x10^{-8}\, e^{-0.02T}\, \frac{cm^3}{s}$	[19,30]
N_{trap}	$5x10^{13}\ \ cm^{-3}$	$5x10^{13}\ \ cm^{-3}$	[18]
β_n	$1x10^{-6}\ \frac{cm^3}{s}$	$1.0x10^{-6}\left(\frac{T}{300}\right)^{-1.9}\ \frac{cm^3}{s}$	[18,31]
β_p	$1x10^{-8}\ \frac{cm^3}{s}$	$1.0x10^{-8}\left(\frac{T}{300}\right)^{-1.9}\ \frac{cm^3}{s}$	[1,12]
S_e, S_p	$2x10^{2}\ \frac{cm}{s}$	$2x10^{2}\ [(1.56x10^{-6})e^{4.186x10^{-2}T} +$<br>$2x10^{2}[\sqrt{T/300}]$	[19,32]
Fermi-Dirac Distribution Function	-	$f(E) = \frac{1}{e^{(E-E_F)/kT} + 1}$	

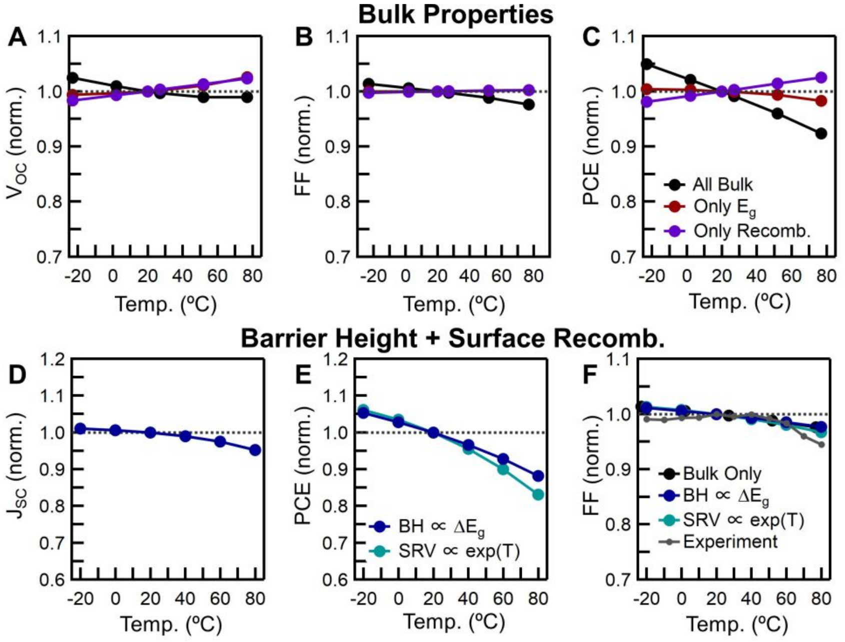

Figure S11: Drift-diffusion modeling of the trends in normalized device performance metrics for triple cation devices. Only bulk perovskite properties are included (ideal contacts) for **A**) V_{OC}, **B**) FF and **C**) PCE. Using non-ideal contacts separating out a change in barrier height proportional to a change in bandgap with a constant surface recombination and a change in the surface recombination proportional to a change in temperatures at a constant barrier height for **D**) J_{SC}, **E**) PCE and **F**) FF.

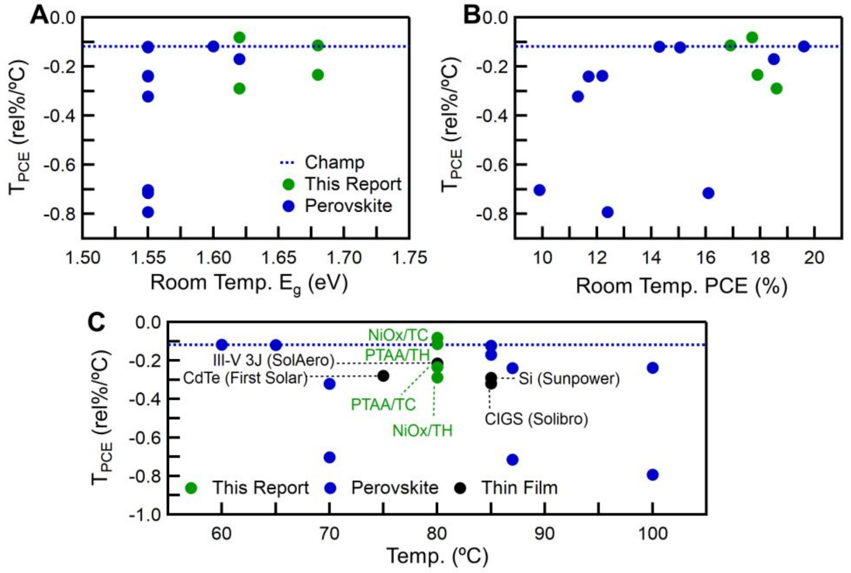

Figure S12: PCE temperature coefficients of literature (blue)[7,20,21,33–39] and this report (green) perovskite solar cells as a function of room temperature **A**) E_g and **B**) PCE. **C**) Relative temperature coefficient for perovskites from literature (blue), this report (green) for Triple Cation (TC) and Triple Halide (TH), and commercialized thin film technologies (black) at the maximum measured operating temperature

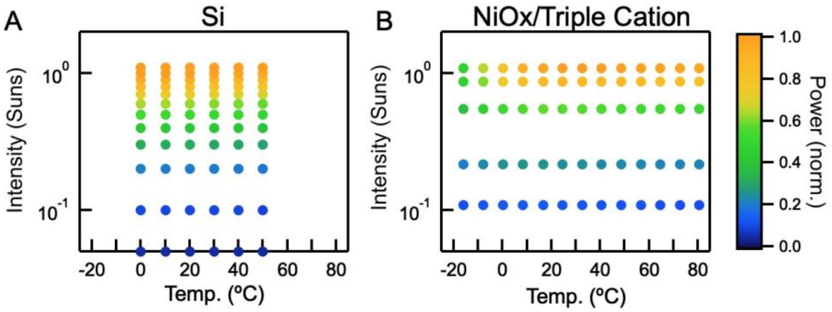

Figure S13: Normalized power versus illumination and temperature for **A)** Si[7] and **B**) NiO_x/Triple Cation with a T_{PCE} of -0.15 rel%/°C and -0.11 rel%/°C, respectively

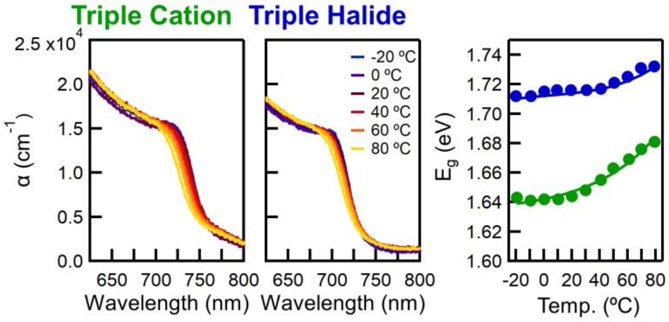

Figure S14: The absorption coefficient, α, for both the perovskites. The band gap was determined using Tauc plots, and an exponential fit was used to extract the rate of change in E_g as a function of temperature.

Table S4: Calculated energy yield for a NiO_x/Triple Cation solar cell and a Si solar cell.

City	Total Irradiance (kWh/m^2)	Module Temp (°C)			Max. Energy Yield (kWh/m^2)	
		Avg. ± Stdev.	Min.	Max.	**Si** 20.4%	**NiO_x/Triple Cation** 17.9%
Minneapolis, MN	1648	13.0 ± 18.7	-23.2	63.7	334	320
Boston, MA	1570	17.1 ± 16.9	-19.9	64.9	315	305
Seattle, WA	1539	17.6 ± 15.9	-6.2	63.8	308	299
Denver, CO	2243	19.6 ± 19.5	-14.2	71	446	425
Albuquerque, NM	2756	25.5 ± 20.1	-9.5	73.2	543	516
Los Angeles, CA	2282	27.2 ± 14.1	5.6	62.4	454	439

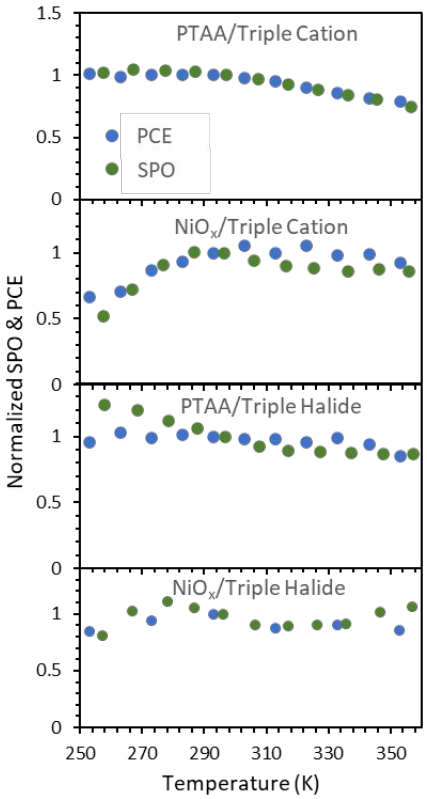

Figure S15: The comparison of the PCE extracted from JV measurements and SPO measurements.

Supplemental References:

(1) Stolterfoht, M.; Wolff, C. M.; Amir, Y.; Paulke, A.; Perdigón-Toro, L.; Caprioglio, P.; Neher, D. Approaching the Fill Factor Shockley-Queisser Limit in Stable, Dopant-Free Triple Cation Perovskite Solar Cells. *Energy Environ. Sci.* **2017**, *10* (6), 1530–1539. https://doi.org/10.1039/c7ee00899f.

(2) Xu, J.; Boyd, C. C.; Yu, Z. J.; Palmstrom, A. F.; Witter, D. J.; Larson, B. W.; France, R. M.; Werner, J.; Harvey, S. P.; Wolf, E. J.; et al. Triple-Halide Wide-Band Gap Perovskites with Suppressed Phase Segregation for Efficient Tandems. *Science (80-.).* **2020**, *367* (6482), 1097–1104. https://doi.org/10.1126/science.aaz4639.

(3) Boyd, C. C.; Shallcross, R. C.; Moot, T.; Kerner, R.; Bertoluzzi, L.; Kavadiya, S.; Chosy, C.; Wolf, E. J.; Werner, J.; James, A.; et al. Overcoming Redox Reactions at Perovskite / Nickel Oxide Interfaces to Boost Voltages in Perovskite Solar Cells. *Joule* **2020**, No. in press, 1–17. https://doi.org/10.1016/j.joule.2020.06.004.

(4) Patel, J. B.; Lin, Q.; Zadvorna, O.; Davies, C. L.; Herz, L. M.; Johnston, M. B. Photocurrent Spectroscopy of Perovskite Solar Cells over a Wide Temperature Range from 15 to 350 K. *J. Phys. Chem. Lett.* **2018**, *9* (1), 263–268. https://doi.org/10.1021/acs.jpclett.7b02935.

(5) Bertoluzzi, L.; Boyd, C. C.; Rolston, N.; Xu, J.; Prasanna, R.; O'Regan, B. C.; McGehee, M. D. Mobile Ion Concentration Measurement and Open-Access Band Diagram Simulation Platform for Halide Perovskite Solar Cells. *Joule* **2020**, *4* (1), 109–127. https://doi.org/10.1016/j.joule.2019.10.003.

(6) National Solar Radiation Databse.

(7) Tress, W.; Domanski, K.; Carlsen, B.; Agarwalla, A.; Alharbi, E. A.; Graetzel, M.; Hagfeldt, A. Performance of Perovskite Solar Cells under Simulated Temperature-Illumination Real-World Operating Conditions. *Nat. Energy* **2019**, *4* (7), 568–574. https://doi.org/10.1038/s41560-019-

0400-8.
(8) Armstrong, S.; Hurley, W. G. A Thermal Model for Photovoltaic Panels under Varying Atmospheric Conditions. *Appl. Therm. Eng.* **2010**, *30* (11–12), 1488–1495. https://doi.org/10.1016/j.applthermaleng.2010.03.012.
(9) Yang, Z.; Surrente, A.; Galkowski, K.; Miyata, A.; Portugall, O.; Sutton, R. J.; Haghighirad, A. A.; Snaith, H. J.; Maude, D. K.; Plochocka, P.; et al. Impact of the Halide Cage on the Electronic Properties of Fully Inorganic Cesium Lead Halide Perovskites. *ACS Energy Lett.* **2017**, *2* (7), 1621–1627. https://doi.org/10.1021/acsenergylett.7b00416.
(10) Dar, M. I.; Jacopin, G.; Meloni, S.; Mattoni, A.; Arora, N.; Boziki, A.; Zakeeruddin, S. M.; Rothlisberger, U.; Grätzel, M. Origin of Unusual Bandgap Shift and Dual Emission in Organic-Inorganic Lead Halide Perovskites. *Sci. Adv.* **2016**, *2* (10). https://doi.org/10.1126/sciadv.1601156.
(11) Ruf, F.; Aygüler, M. F.; Giesbrecht, N.; Rendenbach, B.; Magin, A.; Docampo, P.; Kalt, H.; Hetterich, M. Temperature-Dependent Studies of Exciton Binding Energy and Phase-Transition Suppression in (Cs,FA,MA)Pb(I,Br)3 Perovskites. *APL Mater.* **2019**, *7* (3). https://doi.org/10.1063/1.5083792.
(12) Chen, H. W.; Gulo, D. P.; Chao, Y. C.; Liu, H. L. Characterizing Temperature-Dependent Optical Properties of (MA0.13FA0.87) PbI3 Single Crystals Using Spectroscopic Ellipsometry. *Sci. Rep.* **2019**, *9* (1), 1–9. https://doi.org/10.1038/s41598-019-54636-7.
(13) Brown, C. R.; Eperon, G. E.; Whiteside, V. R.; Sellers, I. R. Potential of High-Stability Perovskite Solar Cells for Low-Intensity–Low-Temperature (LILT) Outer Planetary Space Missions. *ACS Appl. Energy Mater.* **2018**, *2*, 814–821. https://doi.org/10.1021/acsaem.8b01882.
(14) Tan, M.; Chen, B.; Zhang, Y.; Ni, M.; Wang, W. Temperature-Dependent Dynamic Carrier Process of FAPbI 3 Nanocrystals Film. **2020**. https://doi.org/10.1021/acs.jpcc.0c01138.
(15) Kontos, A. G.; Kaltzoglou, A.; Arfanis, M. K.; McCall, K. M.; Stoumpos, C. C.; Wessels, B. W.; Falaras, P.; Kanatzidis, M. G. Dynamic Disorder, Band Gap Widening, and Persistent Near-IR Photoluminescence up to at Least 523 K in ASnI3 Perovskites (A = Cs+, CH3NH3+ and NH2-CHNH2+). *J. Phys. Chem. C* **2018**, *122* (46), 26353–26361. https://doi.org/10.1021/acs.jpcc.8b10218.
(16) O'Donnell, K. P.; Chen, X. Temperature Dependence of Semiconductor Band Gaps. *Appl. Phys. Lett.* **1991**, *58* (25), 2924–2926. https://doi.org/10.1063/1.104723.
(17) Dualeh, A.; Moehl, T.; Nazeeruddin, M. K.; Grätzel, M. Temperature Dependence of Transport Properties of Spiro-MeOTAD as a Hole Transport Material in Solid-State Dye-Sensitized Solar Cells. *ACS Nano* **2013**, *7* (3), 2292–2301. https://doi.org/10.1021/nn4005473.
(18) Landi, G.; Neitzert, H. C.; Barone, C.; Mauro, C.; Lang, F.; Albrecht, S.; Rech, B.; Pagano, S. Correlation between Electronic Defect States Distribution and Device Performance of Perovskite Solar Cells. *Adv. Sci.* **2017**, *4* (10). https://doi.org/10.1002/advs.201700183.
(19) Milot, R. L.; Eperon, G. E.; Snaith, H. J.; Johnston, M. B.; Herz, L. M. Temperature-Dependent Charge-Carrier Dynamics in CH3NH3PbI3 Perovskite Thin Films. *Adv. Funct. Mater.* **2015**, *25* (39), 6218–6227. https://doi.org/10.1002/adfm.201502340.
(20) Jacobsson, T. J.; Tress, W.; Correa-Baena, J. P.; Edvinsson, T.; Hagfeldt, A. Room Temperature as a Goldilocks Environment for CH3NH3PbI3 Perovskite Solar Cells: The Importance of Temperature on Device Performance. *J. Phys. Chem. C* **2016**, *120* (21), 11382–11393. https://doi.org/10.1021/acs.jpcc.6b02858.
(21) Leong, W. L.; Ooi, Z. E.; Sabba, D.; Yi, C.; Zakeeruddin, S. M.; Graetzel, M.; Gordon, J. M.; Katz, E. A.; Mathews, N. Identifying Fundamental Limitations in Halide Perovskite Solar Cells. *Adv. Mater.* **2016**, *28* (12), 2439–2445. https://doi.org/10.1002/adma.201505480.
(22) Van Zeghbroeck, B. Principles of Semiconductor Devices.
(23) Garbuzov, D. Z. Reradiation Effects, Lifetimes and Probabilities of Band-to-Band Transitions in Direct A3B5 Compounds of GaAs Type. *J. Lumin.* **1982**, *27* (1), 109–112. https://doi.org/10.1016/0022-2313(82)90033-3.
(24) Sturge, M. D. Optical Absorption of Gallium Arsenide between 0.6 and 2.75 EV. *Phys. Rev.* **1962**,

127 (3), 768–773. https://doi.org/10.1103/PhysRev.127.768.
(25) Sze, S. M. *Physics of Semiconductor Devices*; John Wiley & Sons, Ltd.: New York, 1981.
(26) Mitonneau, A.; Mircea, A.; Martin, G. M.; Pons, D. Electron and Hole Capture Cross-Sections at Deep Centers in Gallium Arsenide. *Rev. Phys. Appliquée* **1979**, *14* (10), 853–861. https://doi.org/10.1051/rphysap:019790014010085300.
(27) Davies, C. L.; Filip, M. R.; Patel, J. B.; Crothers, T. W.; Verdi, C.; Wright, A. D.; Milot, R. L.; Giustino, F.; Johnston, M. B.; Herz, L. M. Bimolecular Recombination in Methylammonium Lead Triiodide Perovskite Is an Inverse Absorption Process. *Nat. Commun.* **2018**, *9* (1), 1–9. https://doi.org/10.1038/s41467-017-02670-2.
(28) Zhou, Y.; Long, G. Low Density of Conduction and Valence Band States Contribute to the High Open-Circuit Voltage in Perovskite Solar Cells. *J. Phys. Chem. C* **2017**, *121* (3), 1455–1462. https://doi.org/10.1021/acs.jpcc.6b10914.
(29) Futscher, M. H.; Lee, J. M.; McGovern, L.; Muscarella, L. A.; Wang, T.; Haider, M. I.; Fakharuddin, A.; Schmidt-Mende, L.; Ehrler, B. Quantification of Ion Migration in CH3NH3PbI3 Perovskite Solar Cells by Transient Capacitance Measurements. *Mater. Horizons* **2019**, *6* (7), 1497–1503. https://doi.org/10.1039/c9mh00445a.
(30) Zhang, X.; Shen, J. X.; Wang, W.; Van De Walle, C. G. First-Principles Analysis of Radiative Recombination in Lead-Halide Perovskites. *ACS Energy Lett.* **2018**, *3* (10), 2329–2334. https://doi.org/10.1021/acsenergylett.8b01297.
(31) Sherkar, T. S.; Momblona, C.; Gil-Escrig, L.; Ávila, J.; Sessolo, M.; Bolink, H. J.; Koster, L. J. A. Recombination in Perovskite Solar Cells: Significance of Grain Boundaries, Interface Traps, and Defect Ions. *ACS Energy Lett.* **2017**, *2* (5), 1214–1222. https://doi.org/10.1021/acsenergylett.7b00236.
(32) Wang, J.; Fu, W.; Jariwala, S.; Sinha, I.; Jen, A. K.-Y.; Ginger, D. S. Reducing Surface Recombination Velocities at the Electrical Contacts Will Improve Perovskite Photovoltaics. *ACS Energy Lett.* **2019**, *4* (1), 222–227. https://doi.org/10.1021/acsenergylett.8b02058.
(33) Cojocaru, L.; Uchida, S.; Sanehira, Y.; Gonzalez-Pedro, V.; Bisquert, J.; Nakazaki, J.; Kubo, T.; Segawa, H. Temperature Effects on the Photovoltaic Performance of Planar Structure Perovskite Solar Cells. *Chem. Lett.* **2015**, *44* (11), 1557–1559. https://doi.org/10.1246/cl.150781.
(34) Zhang, H.; Qiao, X.; Shen, Y.; Moehl, T.; Zakeeruddin, S. M.; Grätzel, M.; Wang, M. Photovoltaic Behaviour of Lead Methylammonium Triiodide Perovskite Solar Cells down to 80 K. *J. Mater. Chem. A* **2015**, *3* (22), 11762–11767. https://doi.org/10.1039/c5ta02206a.
(35) Schelhas, L. T.; Christians, J. A.; Berry, J. J.; Toney, M. F.; Tassone, C. J.; Luther, J. M.; Stone, K. H. Monitoring a Silent Phase Transition in CH3NH3PbI3 Solar Cells via Operando X-Ray Diffraction. *ACS Energy Lett.* **2016**, *1* (5), 1007–1012. https://doi.org/10.1021/acsenergylett.6b00441.
(36) Bush, K. A.; Bailie, C. D.; Chen, Y.; Bowring, A. R.; Wang, W.; Ma, W.; Leijtens, T.; Moghadam, F.; McGehee, M. D. Thermal and Environmental Stability of Semi-Transparent Perovskite Solar Cells for Tandems Enabled by a Solution-Processed Nanoparticle Buffer Layer and Sputtered ITO Electrode. *Adv. Mater.* **2016**, *28* (201505279), 201505279. https://doi.org/10.1002/adma.201505279.
(37) Fu, F.; Feurer, T.; Weiss, T. P.; Pisoni, S.; Avancini, E.; Andres, C.; Buecheler, S.; Tiwari, A. N. High-Efficiency Inverted Semi-Transparent Planar Perovskite Solar Cells in Substrate Configuration. *Nat. Energy* **2017**, *2* (1), 16190. https://doi.org/10.1038/nenergy.2016.190.
(38) Deng, Y.; Van Brackle, C. H.; Dai, X.; Zhao, J.; Chen, B.; Huang, J. Tailoring Solvent Coordination for High-Speed, Room-Temperature Blading of Perovskite Photovoltaic Films. *Sci. Adv.* **2019**, *5* (12), aax7537. https://doi.org/10.1126/sciadv.aax7537.
(39) Jošt, M.; Lipovšek, B.; Glažar, B.; Al-Ashouri, A.; Brecl, K.; Matič, G.; Magomedov, A.; Getautis, V.; Topič, M.; Albrecht, S. Perovskite Solar Cells Go Outdoors: Field Testing and Temperature Effects on Energy Yield. *Adv. Energy Mater.* **2020**, *10* (25), 2000454. https://doi.org/10.1002/aenm.202000454.
